# Through-Section Characterization of a Large Forged AISI 316 Component: An Industrial Post-Deviation Case Study

**DOI:** 10.3390/ma19214482

**Published:** 2026-10-24

**Authors:** Jude Emele, Aleš Slíva, Mahalingam Nainaragaram Ramasamy, Hasanien Ali Sattar Al Sharif, Jan Dizo

**Affiliations:** 1 Institute of Transport, Faculty of Mechanical Engineering, VSB Technical University of Ostrava, 17. Listopadu 15, 708 00 Ostrava-Poruba, Czech Republic; jude.emele.st@vsb.cz (J.E.); ales.sliva@vsb.cz (A.S.); hasanien.ali.sattar.al.sharif.st@vsb.cz (H.A.S.A.S.); 2 Faculty of Mechanical Engineering, University of Zilina, 17. Univerzitna 8215/1, 010 26 Zilina, Slovakia; jan.dizo@fstroj.uniza.sk

**Keywords:** AISI 316 stainless steel, solution annealing, large-section forging, through-section variation, mechanical properties, intergranular corrosion

## Abstract

This industrial case study examines through-section variations in a 341 mm-diameter AISI 316 stainless-steel component following an unintended extended furnace residence during solution annealing. Instead of being rapidly cooled after the prescribed 1.5 h hold, the component remained in the furnace for approximately 22.25 h, including approximately 16 h at or near 1080 °C. Specimens from the nominal surface, half-radius, and central regions were examined using optical microscopy, tensile and Vickers microhardness testing, SEM–EDS, handheld XRF, and ASTM A262 Practice E testing. Reported mean grain size increased from 286 ± 8 µm at the surface to 304 ± 10 µm at the geometric center, although the underlying uncertainty calculations were unavailable for retrospective verification. Mean yield strength was 36.3% lower in the nominal central-region tensile group (*r*/*R* = 0.23; 231.3 MPa) than in the near-surface group (*r*/*R* = 0.93; 363.3 MPa), while microhardness decreased from 173 to 157 HV0.5. No clear monotonic radial variation was identified in the measured principal alloying elements within the analytical resolution of SEM-EDS and handheld XRF. Post-test visual and SEM examinations revealed no intergranular cracking, grain-boundary separation, or preferential grain-boundary dissolution. Thus, pronounced through-section mechanical-property variation occurred without corresponding detectable compositional variation. Without a conventionally treated control component or direct internal-temperature measurements, the origin of this variation could not be uniquely determined or attributed specifically to the extended furnace residence.

## 1. Introduction

AISI 316 austenitic stainless steel is widely used in transportation and storage equipment, materials-handling systems, marine and coastal installations, and chemical-processing equipment, including tanks, piping, valves, pumps, pressure vessels, and heat exchangers, owing to its favorable combination of mechanical performance, fabricability, and corrosion resistance [1,2,3,4,5]. These properties are strongly influenced by solution annealing, which is commonly conducted at approximately 1050–1100 °C, followed by sufficiently rapid cooling to dissolve chromium-rich carbides, restore a predominantly homogeneous austenitic matrix, and minimize carbide reprecipitation during cooling [6,7]. The resulting microstructure and mechanical properties depend on the annealing temperature, holding time, and subsequent cooling rate, which collectively influence grain growth, recovery, carbide dissolution or reprecipitation, and dislocation structure [8,9,10]. Annealing temperature and holding time strongly influence the microstructure and mechanical response of austenitic stainless steel materials [11,12,13]. Prolonged high-temperature holding can promote recovery, recrystallization, and grain growth, leading to reductions in strength and hardness; the accompanying change in ductility depends on the initial material condition and annealing schedule [13,14,15].

In large-section components, the time required for heat to conduct between the surface and the interior can produce through-section differences in heating and cooling rates. Consequently, different through-section locations may experience distinct thermal histories, even when the imposed external thermal conditions are nominally uniform [8,16,17]. During heating, internal regions may require longer than near-surface regions to approach the furnace temperature, whereas during quenching they may cool more slowly. The combined heating, holding, transfer, and cooling stages can therefore produce location-dependent thermal histories in large-section components. Because internal temperatures of components are rarely monitored directly during industrial processing, the resulting through-section differences in microstructure and mechanical properties may remain undetected.

Beyond possible microstructural changes and mechanical-property variation, corrosion performance is also a critical consideration when austenitic stainless steels undergo high-temperature solution treatment. Although sensitization in austenitic stainless steels generally occurs through chromium-rich carbide (Cr_23_C_6_) precipitation during exposure within the temperature range of approximately 500–850 °C [7,18], rapid solution annealing followed by quenching is generally intended to suppress carbide precipitation and preserve corrosion resistance [6,7,19]. Such sensitized microstructures may exhibit increased susceptibility to intergranular corrosion [20,21,22]. However, the corrosion response of large-section components following excessive residence at the solution-treatment temperature and subsequent rapid quenching remains comparatively underexplored. While sensitization and intergranular corrosion have been extensively investigated under controlled laboratory conditions, relatively few studies have examined the combined microstructural, mechanical, and corrosion behavior of large-section AISI 316 components subjected to industrial heat-treatment overexposure.

One recent qualification approach integrates mechanical testing, microstructural characterization, and complementary evaluation methods to establish structure–property relationships in a large-section industrial AISI 316 component [23]. Despite extensive research on the heat treatment and corrosion behavior of austenitic stainless steels [7,24,25,26], limited information is available on the through-section condition of full-scale wrought AISI 316 components assessed after an industrial solution-treatment residence-time deviation. Furnace-zone records are commonly available, whereas internal material temperatures and resulting radial property variations are rarely measured directly.

In industrial practice, similar heat-treatment residence-time deviations may arise during batch solution annealing of large-section austenitic stainless-steel forgings intended for corrosion-resistant process equipment, including components used in pumps, valves, and pressure-containing assemblies. For example, unexpected interruptions in production operations, temporary unavailability of handling equipment, or delays in quenching-system readiness may prevent the timely extraction of a component following its prescribed holding period. Such circumstances are particularly relevant to large and heavy forgings, for which furnace extraction and transfer to the quenching system require coordinated handling operations. These examples illustrate possible industrial scenarios rather than the documented cause of the deviation investigated in the present study, for which the specific reason for delayed extraction could not be established from the available records.

Accordingly, the present study performs a post-deviation assessment of a large-section AISI 316 component that remained in the furnace for approximately 22.25 h in total, including approximately 16 h at or near the solution-annealing temperature, before quenching. Optical microscopy, tensile testing, Vickers microhardness, SEM–EDS, handheld XRF, and ASTM A262 Practice E testing were used to characterize the surface, half-radius, and center regions of the component. The objectives were to: (i) determine whether measurable radial differences were present; (ii) compare mechanical variation with grain-size and hardness measurements; (iii) assess whether major bulk compositional variation was detectable; and (iv) evaluate susceptibility to intergranular attack under the applied test conditions. Because no conventionally treated control component was available, the study is presented as a post-deviation industrial characterization rather than a controlled determination of whether, or to what extent, the extended residence caused the measured radial differences.

## 2. Materials and Methods

### 2.1. Material Selection and Process

The investigated material was a large-section AISI 316 austenitic stainless-steel component manufactured by forging for a corrosion-resistant process-industry application. The component was supplied in the as-heat-treated condition and had a solid cylindrical geometry with a nominal diameter of 341 mm and an overall length of 5892 mm. To prepare the test material, a 150 mm-long section was first removed from one end of the component. The investigated cross-section used for specimen extraction was subsequently obtained at a location 200 mm from the resulting cut surface. Information regarding the original heat number, detailed prior deformation history, and preceding heat-treatment parameters were not available.

Chemical composition analysis was performed using a Zeiss Ultra 55 field-emission gun scanning electron microscope (FEG-SEM; Zeiss, Oberkochen, Germany) equipped with an Oxford Instruments INCA EDS system (Oxford Instruments, Abingdon, UK). Bulk elemental composition was additionally assessed using a handheld Niton XL2 X-ray fluorescence (XRF) spectrometer (Thermo Fisher Scientific, Waltham, MA, USA). Handheld XRF is well established for the rapid, non-destructive assessment of principal stainless-steel alloying elements, including Cr, Ni, and Mo, as well as for alloy identification and compositional verification [27,28,29]. The measured composition was consistent with the nominal specification of AISI 316 stainless steel, as detailed in Section 3.6.

The material underwent industrial solution annealing using a prescribed heat-treatment schedule consisting of heating to approximately 1080 °C, holding for 1.5 h, and subsequent rapid quenching. The furnace temperature record was analyzed to reconstruct the thermal history of the process. The thermal cycle commenced at approximately 19:09, followed by heating to the solution-annealing temperature range. The furnace reached the nominal solution-annealing temperature of approximately 1080 °C at 01:24. Although the prescribed solution-annealing hold was 1.5 h (scheduled extraction at approximately 02:54), the component remained in the furnace for approximately 22.25 h in total, including approximately 16 h at or near 1080 °C, corresponding to approximately 14.5 h beyond the prescribed 1.5 h hold. The extended furnace residence constituted the documented process deviation associated with the investigated component.

**Figure 1:**
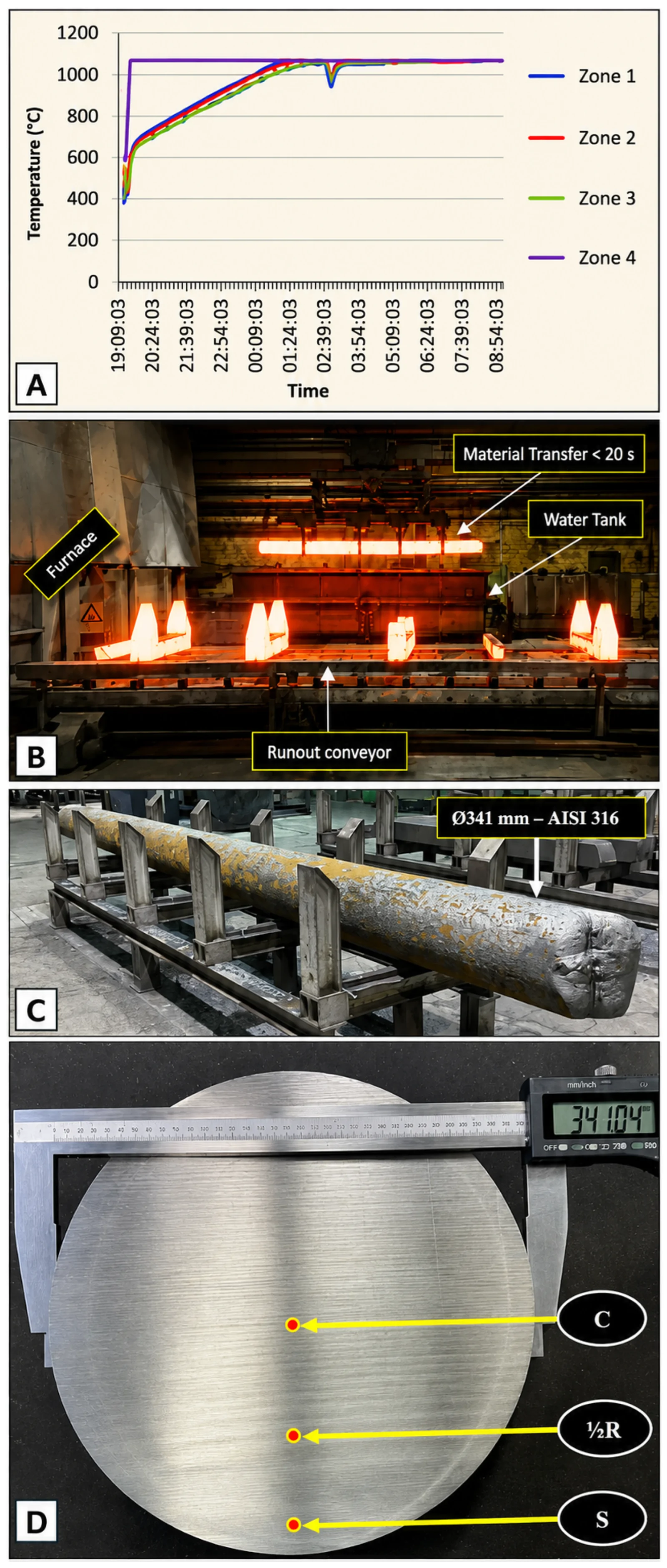
Industrial solution-annealing process and sampling strategy for the Ø341 mm AISI 316 component investigated in the present study: (**A**) archived industrial furnace-zone temperature record surrounding the scheduled extraction time and showing the observed transient temperature fluctuation; (**B**) furnace-to-quench transfer; (**C**) component after the extended furnace residence and subsequent quenching; and (**D**) sampling cross-section showing the nominal surface, half-radius, and central-region sampling groups. The archived temperature record shown in (**A**) does not extend to the eventual component-extraction time.

Following removal from the furnace, the component was transferred to the industrial quench tank in less than 20 s (Figure 1B). The quenching operation was carried out under the plant’s standard production procedure for solution-annealed AISI 316 components. The objective of the rapid quench was to suppress chromium-carbide precipitation during cooling and retain the solution-annealed austenitic microstructure. The original industrial production records did not retain the quench-medium temperature, agitation conditions, or cooling-rate measurements; consequently, these parameters could not be quantified retrospectively.

To characterize spatial variations across the large-section geometry, a radial sampling strategy was adopted. Radial position was expressed using the normalized coordinate *r*/*R*, where *r* is the distance from the geometric center of the component to the center of the tensile-specimen gauge section and *R* is the component radius. For the Ø341 mm component, *R* = 170.5 mm. The sampling groups were nominally designated as the surface (S), half-radius (12R), and central (C) regions. All tensile specimens were oriented longitudinally, parallel to the component axis. The sampling orientations used for the other characterization methods are described in the corresponding subsections. The surface-specimen gauge centers were located 12.5 mm below the external surface (*r* = 158.0 mm), giving an actual normalized coordinate of *r*/*R* = 0.93, which was used in the regression analysis. The half-radius specimens were centered at *r* = 85.25 mm (*r*/*R* = 0.50), whereas the central-region tensile specimens were positioned 40 mm outward from the geometric center. Accordingly, the terms surface, half-radius, and central region are retained as nominal sampling-group designations throughout this study. For tensile testing, the gauge-center coordinate of the nominal central-region group was *r*/*R* = 0.23 because of the specimen geometry, whereas the remaining characterization methods were performed at the geometric center (*r*/*R* = 0).

**Figure 2:**
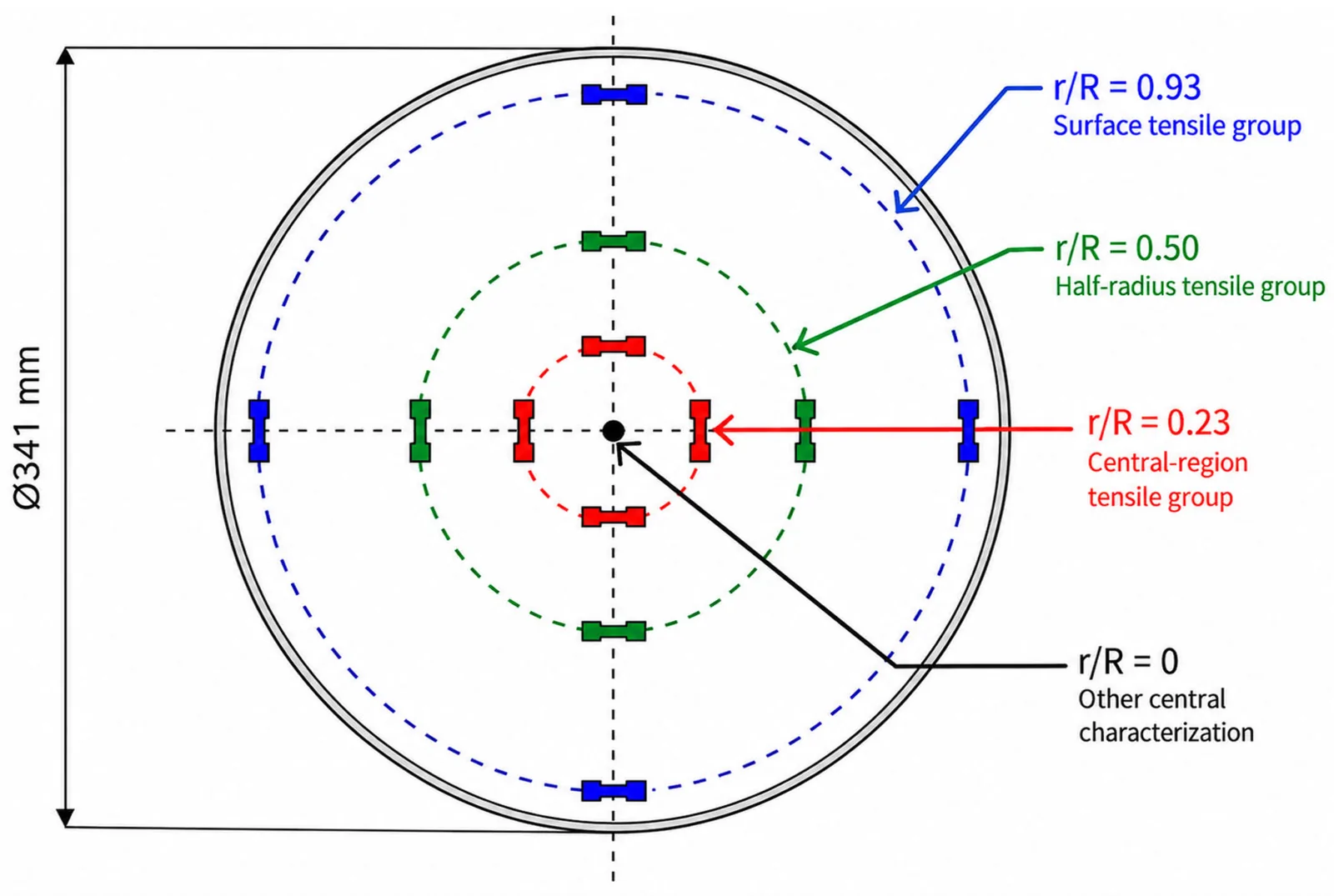
Schematic illustration of the transverse cross-section showing the radial sampling layout of the twelve tensile specimens. The central-region tensile gauge centers were located at *r*/*R* = 0.23, whereas the remaining central-region characterization methods were performed at the geometric center (*r*/*R* = 0).

Four longitudinal tensile specimens were extracted from each radial sampling group. Specimen numbers 1, 2, 3, and 4 correspond to the nominal North, East, South, and West reference orientations, respectively. For the central-region sampling group, the four circumferential directions indicate only the geometric arrangement of the specimens; all four specimens were assigned to the same nominal radial sampling position and therefore do not represent different radial coordinates. To minimize potential end effects, the previously removed 150-mm-long end section was excluded from specimen extraction. The overall thermal history, processing sequence, and sampling strategy are summarized in Figure 1. The detailed radial sampling layout of the twelve longitudinal tensile specimens within the investigated transverse cross-section is illustrated in Figure 2. This sampling strategy enabled the mechanical response to be evaluated across normalized radial positions (*r*/*R*) while accounting for circumferential variability within the component. Accordingly, the relationship between yield strength and *r*/*R* was evaluated using a descriptive linear fit.

### 2.2. Specimen Preparation, Geometry, and Spatial Modeling

Specimens for microstructural, compositional, mechanical, and corrosion characterization were prepared from the three radial positions defined in Section 2.1. As described previously, the investigated cross-section was obtained 200 mm from the cut surface remaining after removal of the 150-mm-long end section, thereby reducing the potential influence of end effects. A photograph of the removed end section is provided in the Supplementary Materials (Figure S1). The relevant specimen-preparation and testing procedures are described below.

#### 2.2.1. Metallographic Preparation and Grain Size Measurement

Metallographic samples were sectioned from the surface (S), half-radius (12R), and center (C) positions using precision cutting to minimize thermal and mechanical damage. Specimens measuring 20 mm × 20 mm were secured in a sample holder, ground and polished using a rotating-disc preparation system in accordance with ASTM E3-11 [30]. Final polishing was performed using colloidal silica. The prepared specimens were subsequently etched using an HCl–HNO_3_ solution to reveal the austenitic grain boundaries. Optical micrographs were acquired at the same magnification using a Leica DM4000 M optical microscope (Leica Microsystems, Wetzlar, Germany).

Grain-size values were obtained from the archived industrial characterization records, in which the measurements were reported as having been performed in accordance with ASTM E112 using the Heyn linear-intercept method [31,32]. One representative optical micrograph was retained for each radial position: surface, half-radius, and center. The archived records reported the corresponding mean grain size, ASTM grain-size number, and an associated uncertainty value for each position. However, the original measurement worksheets were not retained; consequently, the number and total length of the test lines, number of grain-boundary intercepts, the inclusion or exclusion of annealing-twin boundaries during intercept counting, image-calibration procedure, number of independently evaluated fields, and method used to calculate the reported uncertainty values could not be verified retrospectively. The grain-size results are therefore presented as archived values and interpreted descriptively. Moreover, the sample labels, nominal radial-position designations, and actual tensile gauge-center coordinates are provided in Table 1.

#### 2.2.2. SEM–EDS and XRF Chemical Analysis

**Figure 3:**
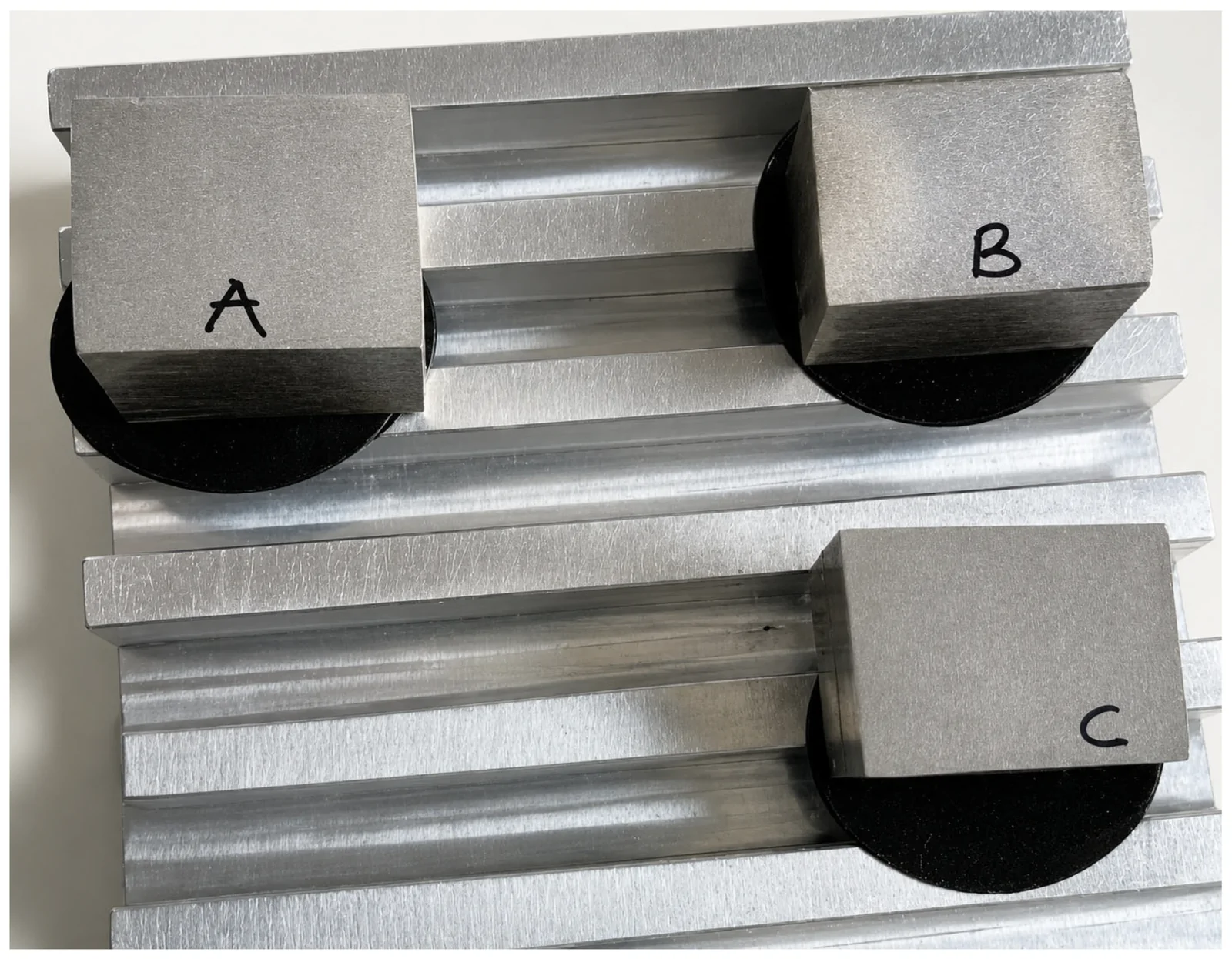
Representative specimens prepared for SEM–EDS compositional characterization from the nominal (**A**) surface, (**B**) half-radius, and (**C**) central-region sampling groups of the Ø341 mm AISI 316 stainless-steel component.

Chemical composition was evaluated using scanning electron microscopy coupled with energy-dispersive X-ray spectroscopy (Zeiss Ultra 55 FEG-SEM, Oberkochen, Germany) and handheld X-ray fluorescence (Niton XL2, Thermo Fisher Scientific, Waltham, MA, USA). Representative specimens prepared for SEM–EDS compositional characterization from the surface, half-radius, and central-region sampling groups are shown in Figure 3. SEM–EDS point analyses provided localized elemental information, whereas handheld XRF provided semi-quantitative bulk compositional measurements, allowing complementary assessment at the corresponding radial positions. The archived records did not retain the number of replicate measurements or the calculation basis of the reported SEM–EDS uncertainties.

Representative SEM–EDS spectra and handheld XRF measurements were used to compare the principal alloying elements among the three sampling groups. The concentrations of Cr, Ni, Mo, and Mn were examined for systematic differences with radial position. The compositional assessment was intended to determine whether any observed differences exceeded the analytical resolution and repeatability of the applied techniques, rather than to establish certified quantitative elemental concentrations or detailed compositional gradients across the component.

Fe was treated as the balance, whereas carbon was not quantified by handheld XRF because its low atomic number is outside the reliable analytical range of the instrument. Carbon values obtained by SEM–EDS, when present in the spectra, were not used for quantitative comparison because they are sensitive to surface contamination and specimen-preparation conditions. Consequently, only differences clearly exceeding the expected analytical variability of the respective techniques were regarded as analytically distinguishable. Additional representative SEM–EDS spectra supporting the compositional assessment are provided in the Supplementary Materials (Figure S2).

#### 2.2.3. Tensile Specimens and Mechanical Testing

The archived industrial test records identified ASTM E8/E8M-22 [33] as the tensile-testing standard applied by the testing laboratory. The round tensile specimens had the same nominal geometry, with an initial diameter (*d*_0_) of 10 mm, a parallel length (*L_c_*) of 75 mm, a gauge length (*L*_0_) of 50 mm, and an initial cross-sectional area (*A*_0_) of 78.5 mm^2^. The specimen geometry and principal dimensions are presented in Figure S3 in the Supplementary Materials. The specimens were assigned to three nominal radial sampling groups: surface (S), half-radius (12R), and central region (C). The component radius, (*R*), was 170.5 mm. The surface-group gauge centers were located 12.5 mm beneath the external surface, corresponding to (*r* = 158.0) mm and (*r*/*R* = 0.93). The half-radius gauge centers were located at (*r* = 85.25) mm, corresponding to (*r*/*R* = 0.50). Unlike the metallographic, compositional, microhardness, and corrosion specimens obtained at the geometric center (*r*/*R* = 0), the tensile-specimen gauge centers in the nominal central-region group were located at a radial distance of 40 mm from the component axis, corresponding to (*r*/*R* = 0.23). This offset arose from the specimen geometry and extraction arrangement and was accounted for consistently in the radial regression analysis.

Four longitudinal tensile specimens were tested from each sampling group, giving a total of 12 specimens. Specimen numbers 1–4 corresponded to the nominal North, East, South, and West orientations, respectively. For the surface and half-radius groups, these designations identify the circumferential sampling directions. For the central-region group, they describe the arrangement of the four specimens at the same nominal radial coordinate and do not represent independent radial sampling levels. Accordingly, the four specimens within each group were treated as spatial replicates of the corresponding radial position.

Mechanical testing was conducted at room temperature using a Shimadzu AGS-X universal testing machine with a rated frame capacity of 250 kN (Shimadzu Corporation, Kyoto, Japan). Test control and data acquisition were performed using LABMASTER (Version 2.5.3.21, Hegewald & Peschke Meß-und Prüftechnik GmbH, Nossen, Germany). The measured properties were the 0.2% proof stress (yield strength, *σ_y_*), ultimate tensile strength (*σ_UTS_*), and elongation after fracture (*A*). Although the testing machine and laboratory method were identified from the original industrial records, the archived documentation did not retain the installed load-cell capacity, applied strain rate, crosshead speed, or extensometer configuration. These test parameters could therefore not be reported retrospectively.

#### 2.2.4. Vickers Microhardness Testing

Vickers microhardness measurements were performed at the surface, half-radius, and center regions to characterize the hardness distribution in the investigated heat-treated component. Measurements were carried out using a laboratory microhardness tester in accordance with ASTM E384-22 [34] under a load of 0.5 kgf (HV0.5) with 40× optical magnification. Indentation sites were selected to avoid preparation artefacts, grain-boundary intersections, and edge effects. Three indentations were made at each radial position, and the archived records contained only the instrument-displayed aggregate HV0.5 value for each radial position. Individual indentation values and associated uncertainty measures were unavailable. The retained raw Vickers microhardness measurement records are provided in the Supplementary Materials (Figure S5).

#### 2.2.5. ASTM A262 Practice E (Strauss) Intergranular Corrosion Testing

Rectangular corrosion-test specimens (approximately 100 mm × 20 mm × 4 mm) were machined from the surface (S), half-radius (12R), and center (C) regions of the Ø341 mm AISI 316 stainless-steel component to evaluate susceptibility to sensitization-induced intergranular corrosion in accordance with ASTM A262 Practice E (Strauss test) [35]. One corrosion-test specimen was evaluated from each radial position, giving a total of three specimens. The test solution was prepared by dissolving 100 g of reagent-grade copper sulfate pentahydrate (CuSO_4_·5H_2_O) in 500 mL of distilled water, followed by the addition of 100 mL of concentrated sulfuric acid (specific gravity 1.84) and dilution with distilled water to a total volume of 1000 mL. Electrolytic-grade metallic copper shot (grindings) were added in sufficient quantity to maintain contact with the specimen surfaces throughout the test.

**Figure 4:**
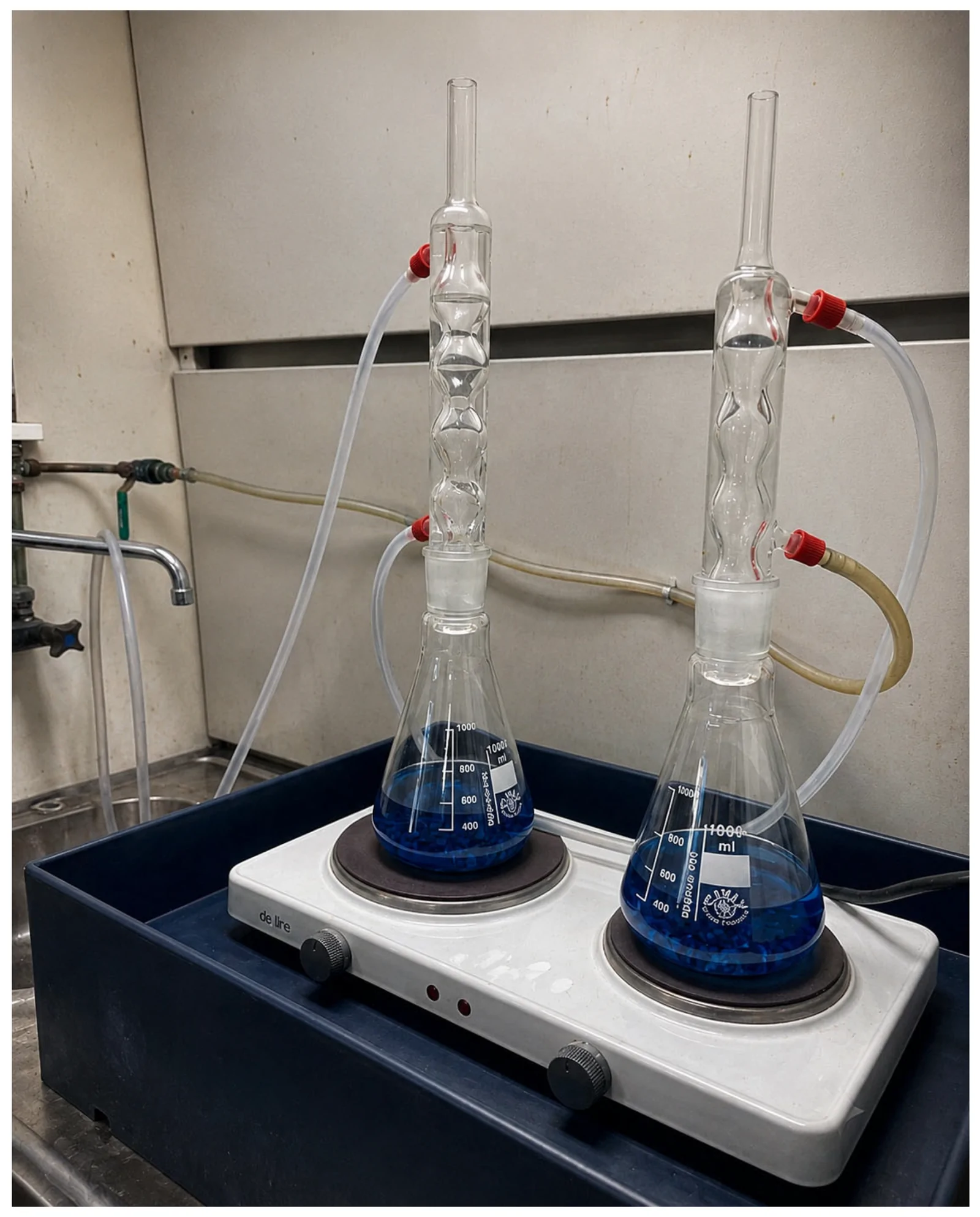
Experimental setup for ASTM A262 Practice E (Strauss) intergranular corrosion testing of rectangular corrosion-test specimens in boiling sulfuric acid-copper sulfate solution using 1000 mL Erlenmeyer flasks fitted with reflux condensers.

**Figure 5:**
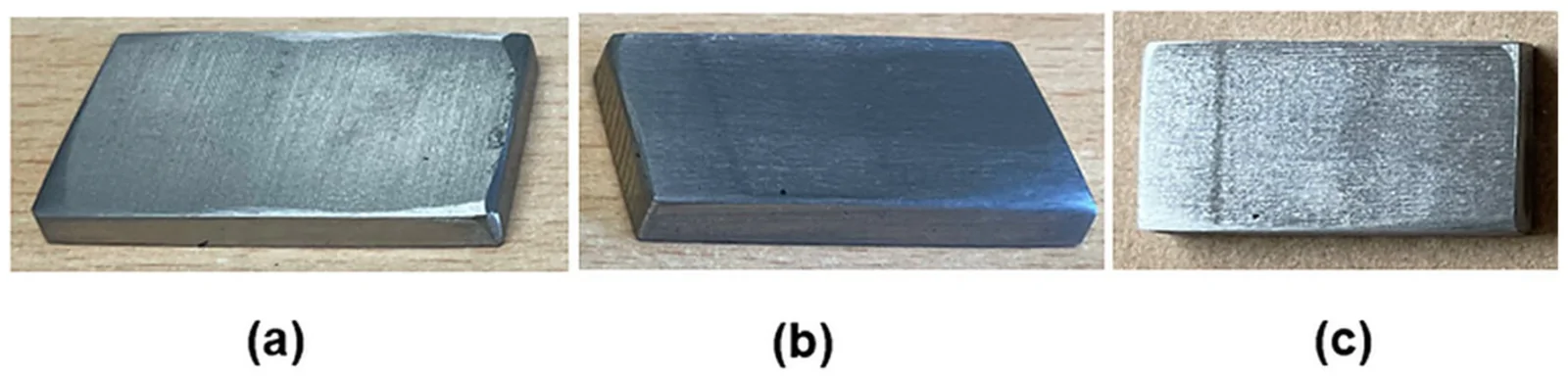
Representative rectangular corrosion-test specimens extracted from the (**a**) surface (S), (**b**) half-radius (12R), and (**c**) center (C) regions for intergranular corrosion testing according to ASTM A262 Practice E (Strauss test).

The specimens were exposed under continuous boiling reflux for approximately 22 h (Figure 4). This duration exceeded the 15 h minimum exposure specified for ASTM A262 Practice E and is therefore reported explicitly. Following exposure, the specimens were bent through 180° over a mandrel having a diameter equal to the specimen thickness in accordance with ASTM A262 Practice E. The external tensile surface of each bent specimen was examined visually and under low magnification, nominally 5–20×. Where the result was uncertain, metallographic examination of a longitudinal section through the outer bend radius was performed at 100–250×. The acceptance criterion was the absence of intergranular fissuring or cracking. Edge cracking and deformation markings were not considered evidence of intergranular attack unless associated with intergranular fissuring. Representative corrosion-test specimens before ASTM A262 Practice E testing are shown in Figure 5.

After exposure according to ASTM A262 Practice E, the specimens were characterized by SEM. Representative regions of the bent surfaces were examined using secondary-electron imaging (SE2) at an accelerating voltage of 20 kV. Images were acquired at magnifications of 1000× and 2000×, with working distances of approximately 26–29 mm. The examination focused on identifying evidence of intergranular cracking, grain-boundary separation, preferential grain-boundary dissolution, and deformation-related surface features.

#### 2.2.6. Descriptive Linear Analysis of Mean Yield Strength Across Radial Levels

To describe the sampled radial variation in mean yield strength, the actual tensile gauge-center coordinate, *r*/*R*, was used as the explanatory coordinate. The three unique radial levels were: *r*/*R* = 0.23 for the central-region group, *r*/*R* = 0.50 for the half-radius group, and *r*/*R* = 0.93 for the surface group. The four tensile specimens tested at each coordinate were first summarized by their group mean and standard deviation. A descriptive linear fit was then applied to the three group means rather than to the 12 individual specimen measurements:

$${\bar{\sigma }}_{y,j}=\beta_{0}+\beta_{1}{\left(r/R\right)}_{j}+\varepsilon_{j}$$

where:

*j* = 1, 2, 3 denotes the three radial levels.

${\bar{\sigma }}_{y,j}$ is the mean yield strength at radial level *j*

*β*_0_ is the intercept

*β*_1_ is the descriptive slope representing the change in mean yield strength per unit change in *r*/*R* across the sampled levels, and

*ε_j_* is the deviation of the observed group mean from the fitted line.

**Figure 6:**
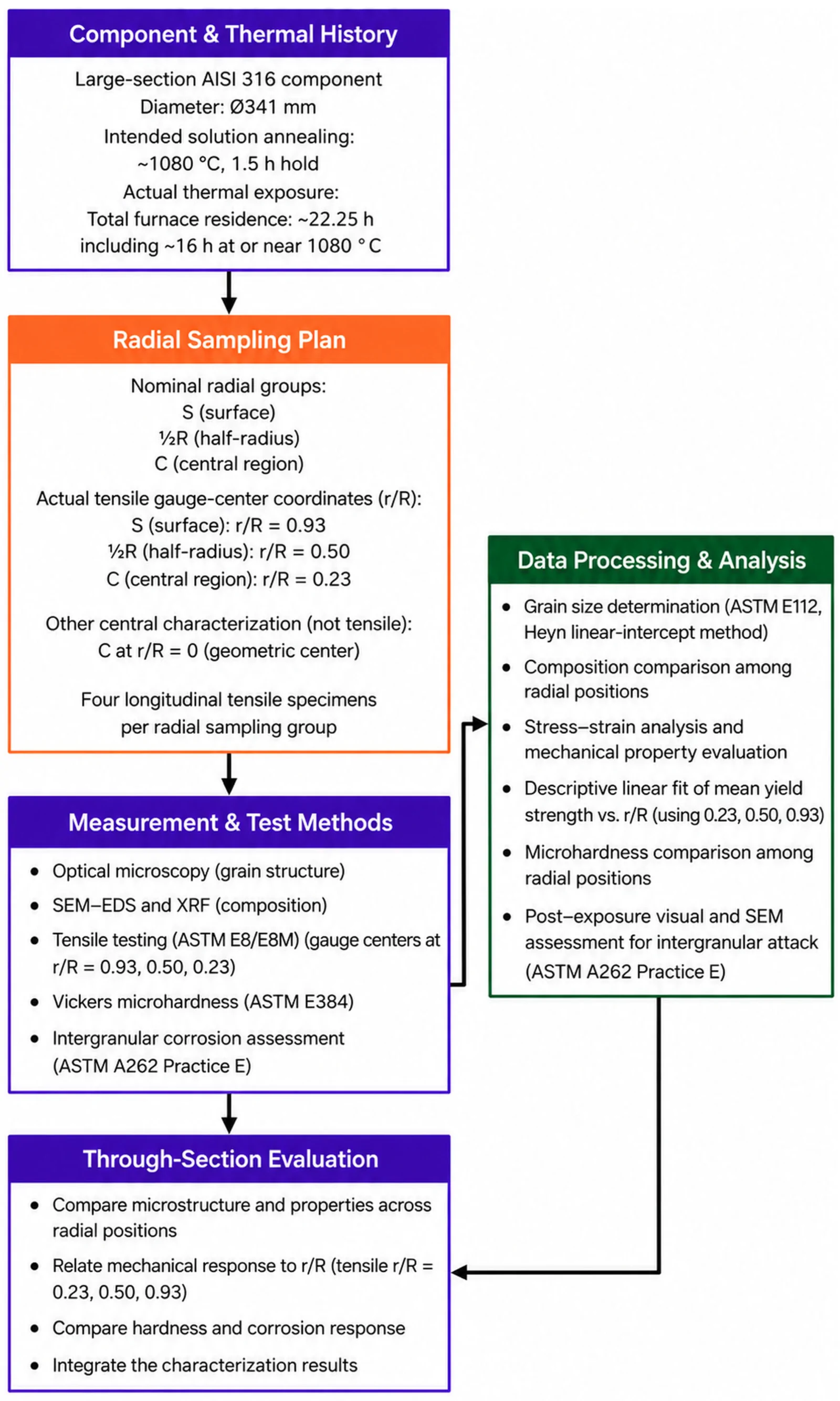
Experimental and analytical workflow for the post-deviation through-section characterization of the large-section AISI 316 component.

Because only three unique radial coordinates were investigated, the linear analysis was restricted to the three radial-group means. The four tensile specimens at each coordinate were treated as within-position spatial replicates and used to calculate the corresponding mean and standard deviation, rather than as independent radial levels. Accordingly, the fitted slope and coefficient of determination were used only to describe the variation among the sampled radial-group means. No statistically validated continuous radial relationship is inferred, because the fit was based on only three observations and one residual degree of freedom. The experimental and analytical workflow, from component thermal history and radial sampling through characterization, data processing, and integrated through-section evaluation, is summarized in Figure 6.

## 3. Results

### 3.1. Thermal History and Process Deviation

The furnace record indicated that all monitored furnace zones reached approximately 1080 °C and remained closely aligned throughout the thermal cycle. Although a transient fluctuation occurred near the scheduled extraction time (Figure 1A), no sustained divergence among the monitored furnace-zone temperatures was observed. The documented process deviation consisted of delayed extraction of the component. Consequently, the component remained in the furnace for approximately 22.25 h in total, including approximately 16 h at or near 1080 °C, corresponding to approximately 14.5 h beyond the prescribed 1.5 h hold. The recorded temperatures represent furnace-zone measurements only and do not provide direct information on the transient temperature distribution within the component. Accordingly, the furnace record confirms the thermal conditions within the monitored furnace zones but does not establish the internal temperature distribution or through-section thermal history of the component during processing.

### 3.2. Microstructural Characteristics and Radial Grain-Size Distribution

**Figure 7:**
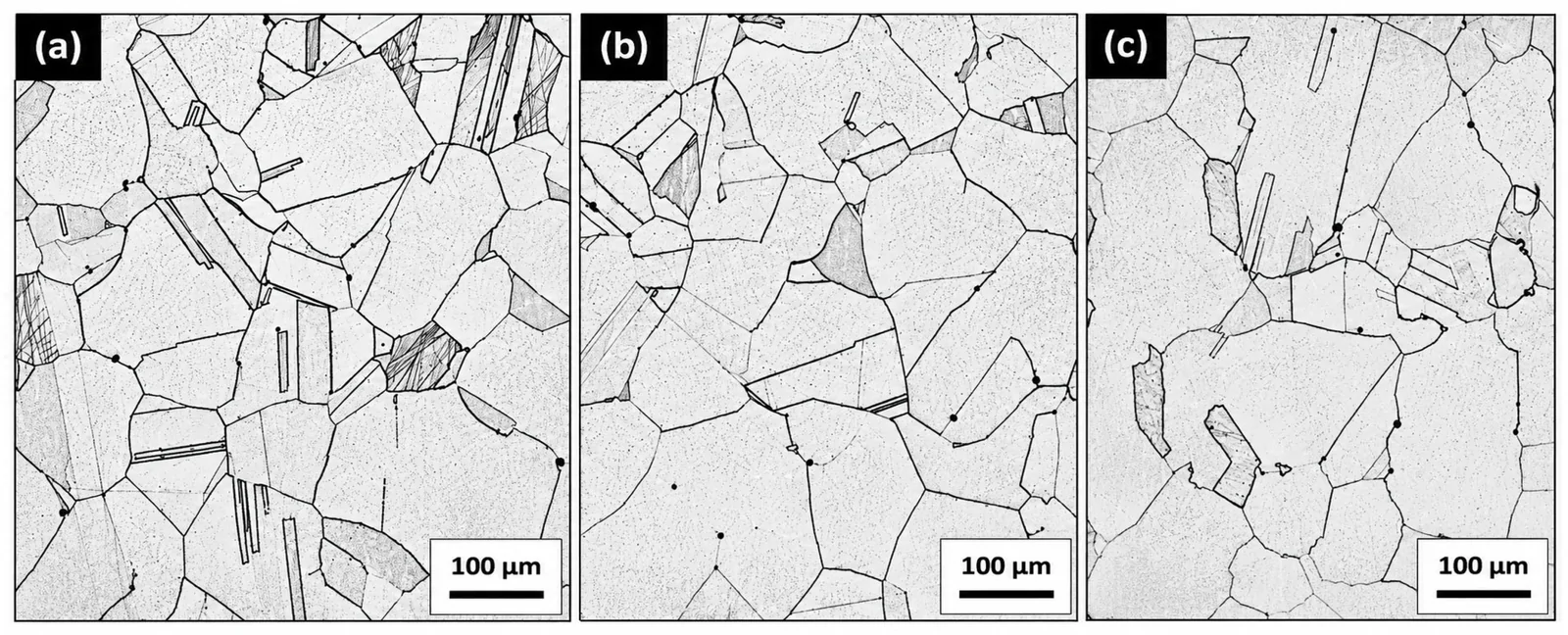
Optical micrographs of specimens extracted from the (**a**) surface, (**b**) half-radius, and (**c**) center regions of the investigated large-section AISI 316 stainless-steel component. Annealing twins are visible within the austenitic matrix. Scale bar = 100 µm.

Optical micrographs from the surface, half-radius, and center regions (Figure 7) exhibited broadly similar equiaxed austenitic grain morphologies containing characteristic annealing twins. Representative micrographs suggested a modest reduction in the apparent frequency of grain-boundary intersections from the surface toward the center, qualitatively consistent with the archived grain-size results. Small dark features were observed at all three radial positions. Because these features were not chemically or crystallographically characterized, their identity could not be established in the present study. No systematic difference in their occurrence was observed among the representative metallographic fields, and they may represent non-metallic inclusions, particle pull-out during specimen preparation, or other metallographic artefacts.

The reported mean grain size increased from 286 µm at the surface to 292 µm at the half-radius position and 304 µm at the center. The corresponding reported ASTM grain-size number (*G*) decreased from 0.70 at the surface to 0.52 at the center. The archived records also provided associated uncertainty values of ±8, ±7, and ±10 µm, respectively. Because the underlying intercept counts, field-level measurements, and uncertainty calculations were unavailable, these values were not independently recalculated or subjected to additional statistical analysis. Accordingly, the archived results indicate a modest descriptive increase in reported grain size toward the component center. The archived grain-size results are summarized in Table 2.

### 3.3. Tensile Behavior and Through-Section Mechanical Variation

Mean yield strength decreased from 363.3 ± 21.7 MPa in the surface sampling group (*r*/*R* = 0.93) to 231.3 ± 2.4 MPa in the central-region sampling group (*r*/*R* = 0.23), based on four specimens per group. Mean ultimate tensile strength decreased from 616.3 ± 2.8 MPa at the surface to 505.5 ± 6.4 MPa at the central-region sampling group, whereas the half-radius sampling group exhibited intermediate mean strength values and greater variability in ultimate tensile strength, as reflected by a standard deviation of 47.4 MPa. Overall, progressively lower mean yield-strength values were measured from the surface group toward the central-region group. Mean ultimate tensile strength also decreased across the three radial sampling groups; however, substantial within-group scatter was observed at the half-radius location.

The half-radius sampling group exhibited two comparatively low ultimate tensile strength (UTS) values (529 and 533 MPa) and two higher values (596 and 625 MPa). Review of the available specimen dimensions, fracture locations, and tensile-test records did not identify any documented experimental anomaly, specimen-preparation defect, or testing irregularity that could explain this variation. The observed scatter may reflect local microstructural heterogeneity within the half-radius region, potentially associated with variations in prior forging deformation or local recovery and recrystallization state. However, these possible contributions were not directly characterized, and the origin of the UTS scatter remains unresolved.

Furthermore, the four circumferential measurements were insufficient to establish a systematic circumferential trend.

The individual tensile-test results for specimens extracted from the three radial sampling groups are summarized in Table 3. The four specimens tested at each radial level were used to calculate the corresponding mean yield strength and standard deviation for the descriptive regression analysis. These group statistics, together with the fitted values and residuals of the descriptive linear fit, are presented in Table 4. Mean elongation showed a monotonic increase across the sampled radial positions, from 56.0 ± 1.4% at the surface to 57.8 ± 2.6% at the half-radius position and 62.8 ± 1.0% in the central-region group. Although the measured values indicate greater ductility toward the component interior, the limited number of radial sampling levels and absence of a conventionally treated control component require this trend to be interpreted descriptively.

**Figure 8:**
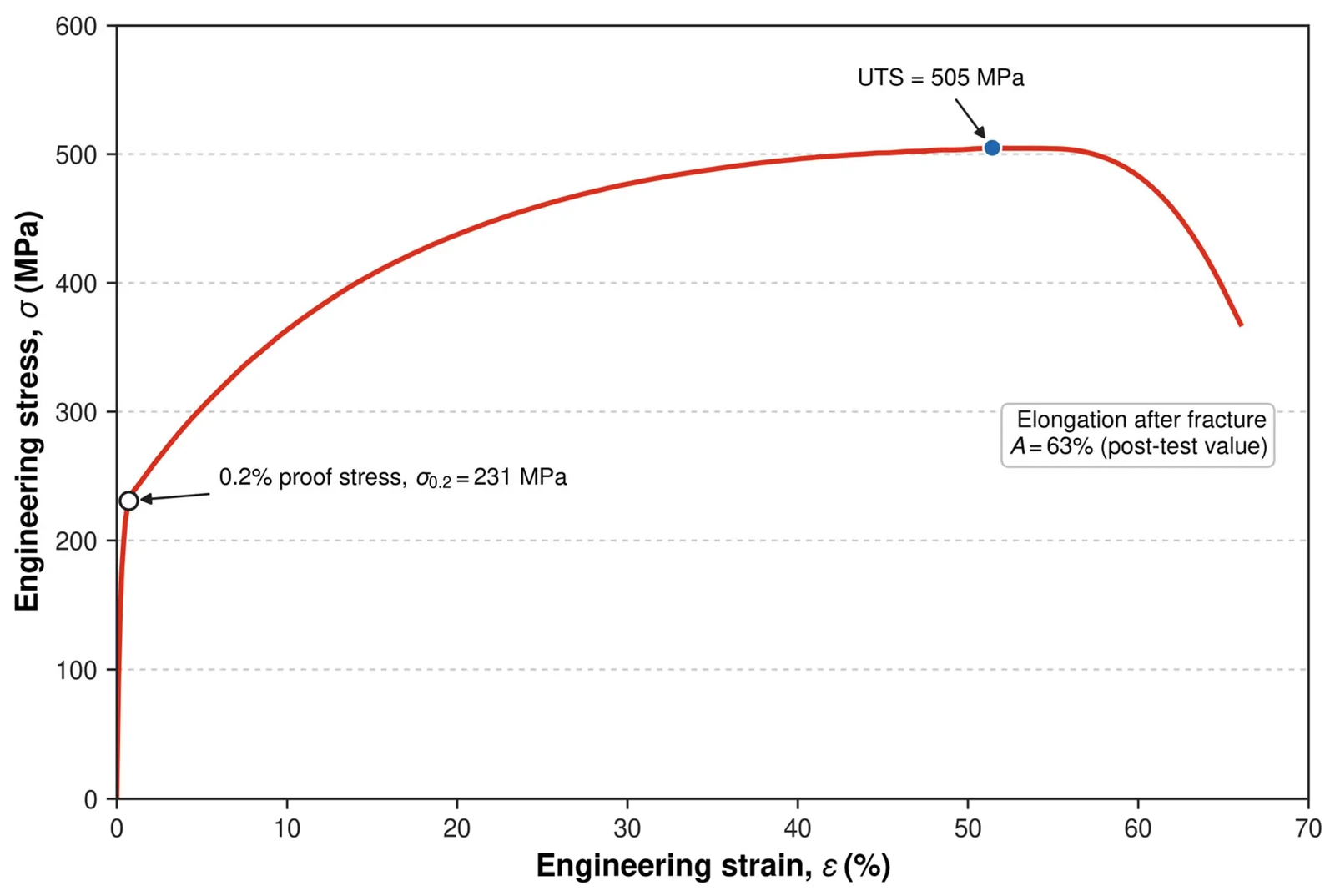
Engineering stress–strain curve of specimen C1 extracted from the central region (*r*/*R* = 0.23). The graph was digitally redrawn from the original tensile-test data to improve graphical clarity while preserving the original experimental measurements.

Figure 8 presents the representative engineering stress–strain response of specimen C1 extracted from the central-region sampling group (*r*/*R* = 0.23). The corresponding tensile properties were a yield strength of 231 MPa, an ultimate tensile strength of 505 MPa, and an elongation after fracture of 63%. Original machine-generated plots were recoverable for six of the 12 tested specimens and are provided in the Supplementary Materials (Figure S4).

**Figure 9:**
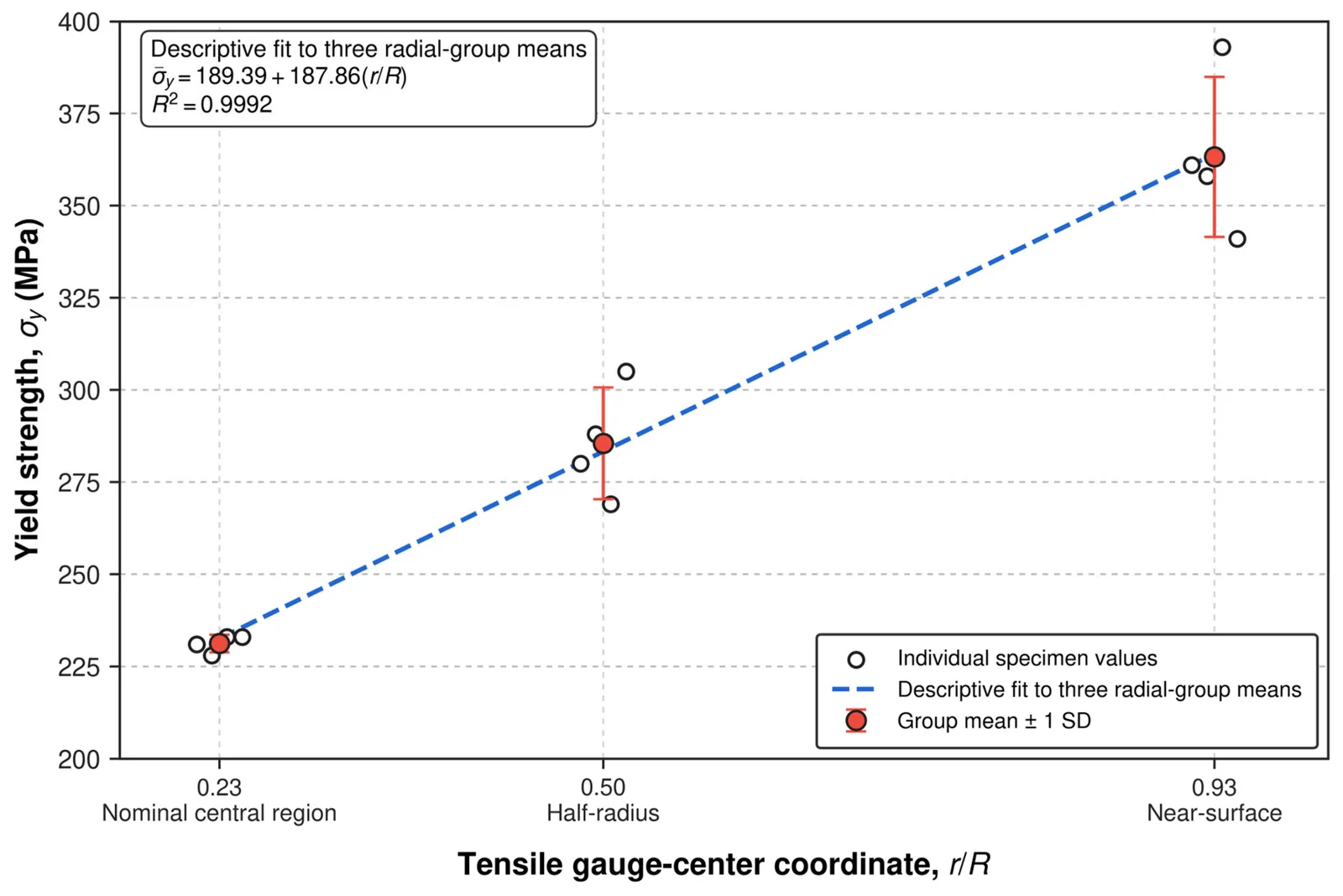
Yield strength as a function of the actual normalized radial coordinate of the tensile-specimen gauge center, *r*/*R*. Open circles represent the four individual specimen measurements at each radial coordinate. Orange-filled circles and error bars indicate the corresponding group mean ± one standard deviation (SD). The dashed line represents a descriptive linear fit to the three radial-group means at *r*/*R* = 0.23, 0.50, and 0.93. Individual specimen measurements were treated as within-position spatial replicates and not as independent radial levels in the regression.

Section 3.4 provides a first-order descriptive fit to the sampled radial trend, whereas Figure 9 shows the measured yield-strength differences among the three tensile gauge-center coordinates, *r*/*R* = 0.23, 0.50, and 0.93.

### 3.4. Descriptive Linear Analysis of Mean Yield Strength Across the Three Radial Levels

Using the three radial-group mean yield-strength values at *r*/*R* = 0.23, 0.50, and 0.93, the descriptive first-order relationship was:

$${\bar{\sigma }}_{y}=189.39+187.86(r/R),\;\mathrm{with}\;R^{2}=0.9992$$

where: ${\bar{\sigma }}_{y}$ is expressed in MPa. The fitted slope corresponds to an increase of approximately 187.9 MPa in mean yield strength per unit increase in *r*/*R* across the three sampled radial levels. The high coefficient of determination indicates that the three group means were closely aligned with a straight line. However, because the fit was based on only three radial levels and had one residual degree of freedom, it should be interpreted solely as a descriptive summary of the sampled means and not as a statistically validated continuous radial relationship. The four specimens within each radial group were retained in the graphical presentation and summary statistics to show within-position spatial variability. They were not treated as 12 independent observations of the radial coordinate in the fitted model.

**Figure 10:**
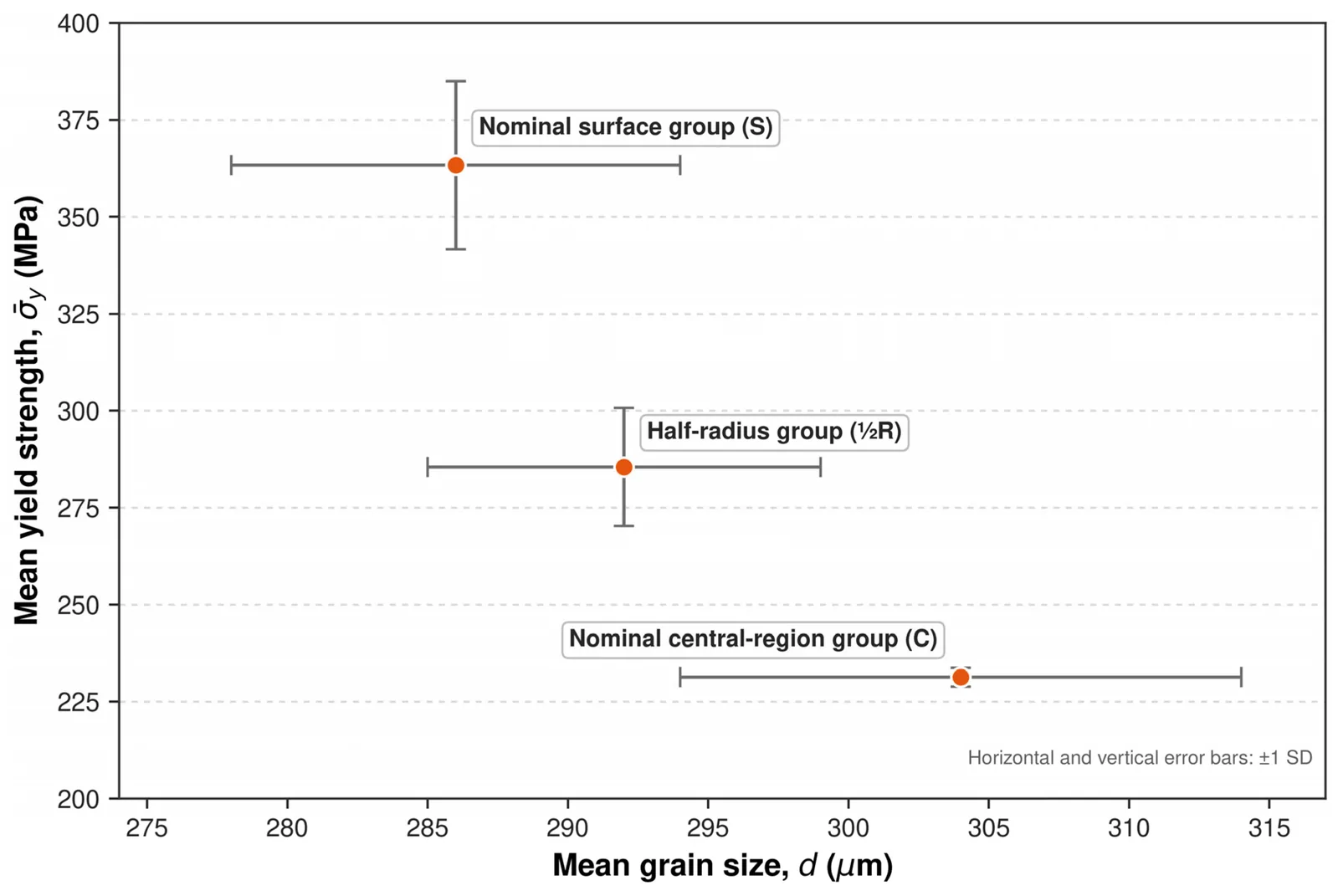
Descriptive comparison of measured mean grain size and mean yield strength across the three nominal radial groups. Error bars represent ±1 SD. Central-region tensile and metallographic measurements were obtained at (*r*/*R* = 0.23) and (*r*/*R* = 0), respectively; therefore, the figure does not represent a pointwise or causal structure–property correlation.

As shown in Figure 10, lower mean yield-strength values at smaller *r*/*R* were accompanied by higher measured mean grain sizes. The three sampled radial groups therefore exhibited an inverse correspondence between mean grain size and mean yield strength; however, this comparison does not establish that grain-size variation caused the strength difference.

### 3.5. Fracture Surface Morphology

**Figure 11:**
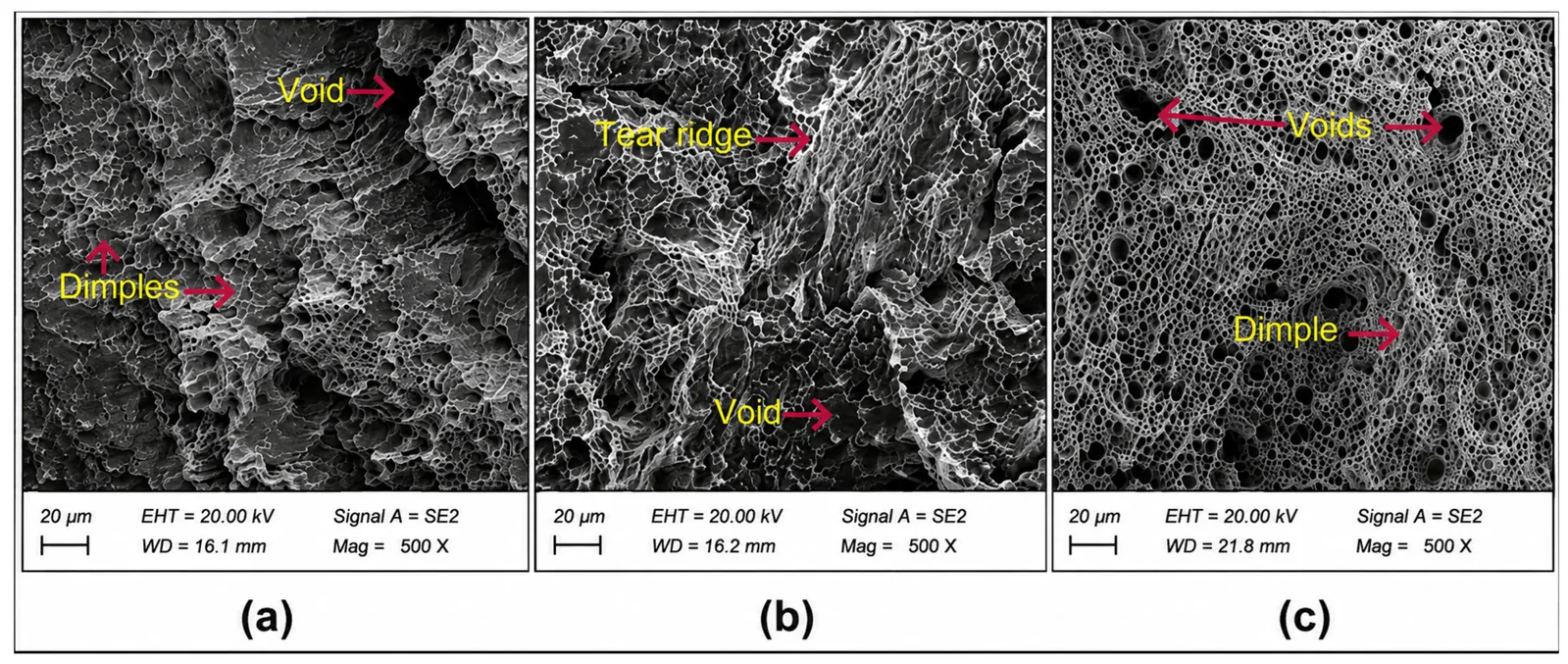
Representative SEM fractographs of tensile fracture surfaces from specimens extracted from (**a**) the surface (S1, *r*/*R* = 0.93), (**b**) the half-radius position (R1, *r*/*R* = 0.50), and (**c**) the central region (C1, *r*/*R* = 0.23). All images were acquired using secondary-electron (SE2) imaging at 500× magnification. Scale bar: 20 μm.

Representative SEM fractographs of tensile specimens from the surface (S1), half-radius (R1), and central-region (C1) groups are presented in Figure 11. All three fracture surfaces exhibited predominantly ductile fracture characterized by extensive microvoid coalescence, dimple formation, and well-developed tear ridges. Larger voids surrounded by finer dimpled features were observed throughout the fracture surfaces, consistent with substantial plastic deformation prior to final fracture.

Despite local variations in dimple size, void distribution, and plastic-flow features, the overall fracture morphology was similar among the three representative specimens. No cleavage facets, river patterns, or intergranular fracture features were observed in the examined fields. These observations indicate that the dominant fracture mechanism remained ductile across the investigated radial groups.

### 3.6. Radial Chemical-Composition Comparison

**Figure 12:**
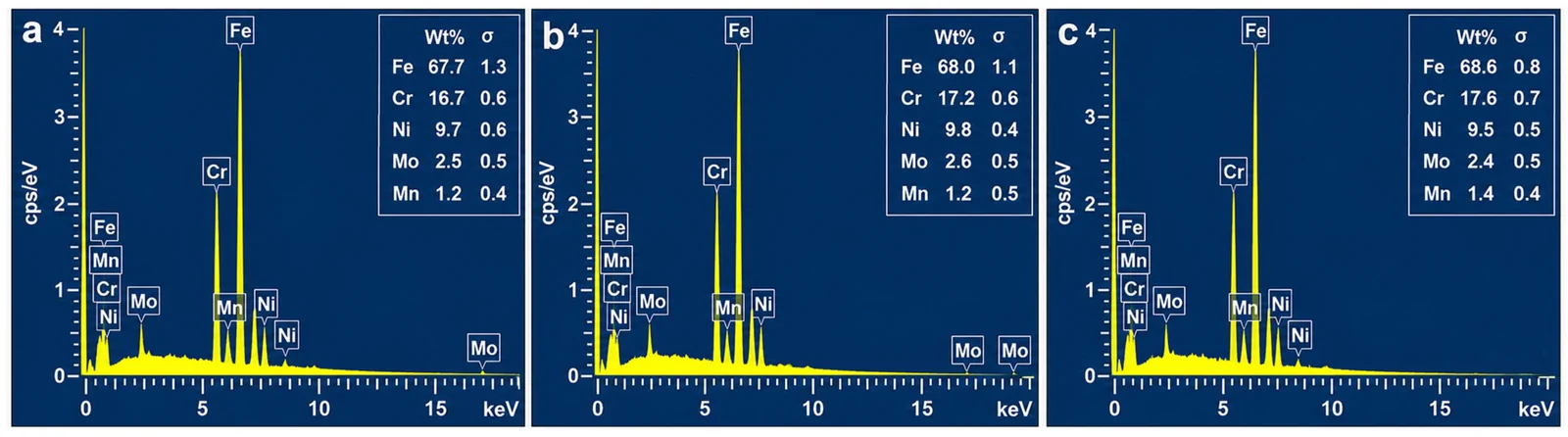
Representative SEM–EDS spectra acquired from the (**a**) surface, (**b**) half-radius, and (**c**) center regions of the large-section AISI 316 stainless-steel component.

**Figure 13:**
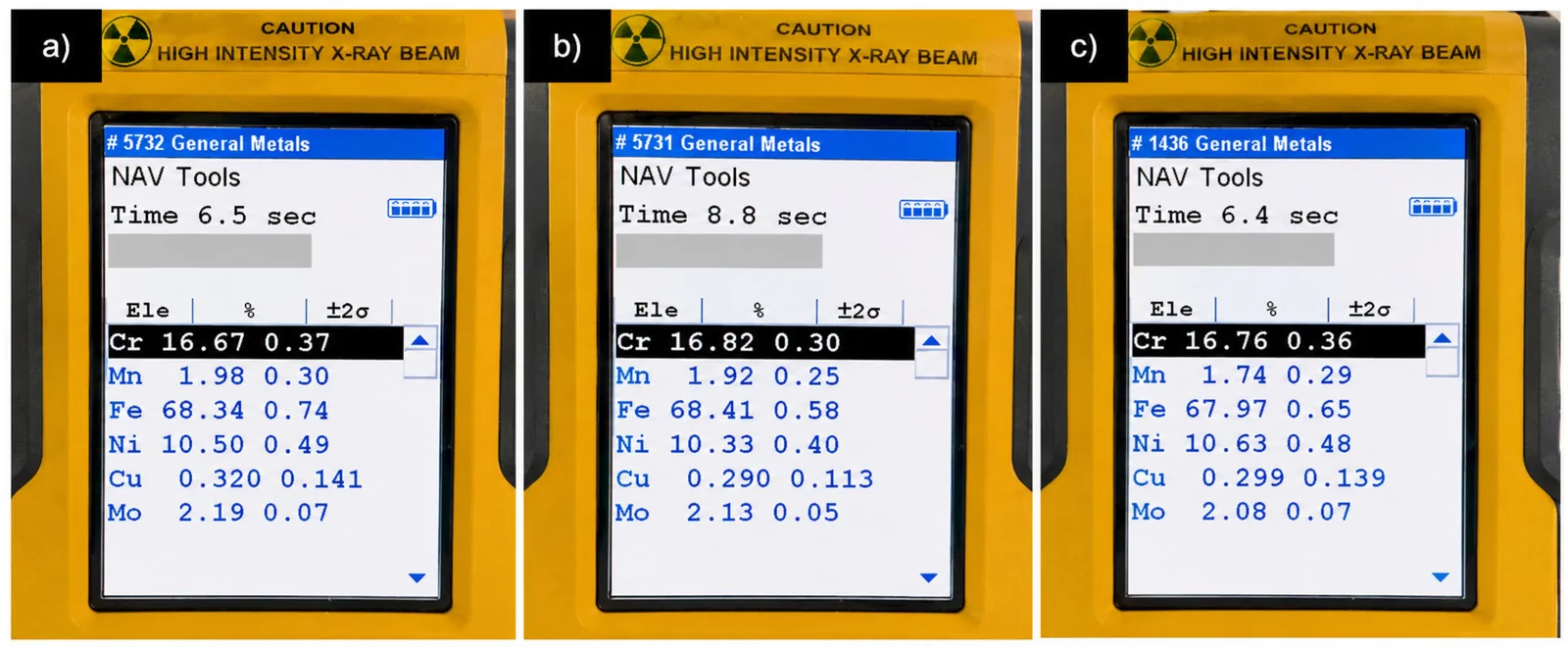
Representative handheld XRF elemental-analysis readouts: (**a**) surface, (**b**) half-radius, and (**c**) center regions of the large-section AISI 316 stainless-steel component.

The SEM–EDS spectra from the three radial locations were comparable and were dominated by Fe, Cr, Ni, Mn, and Mo peaks (Figure 12). Additional representative spectra are provided in the Supplementary Materials (Figure S2). Handheld XRF measurements likewise showed only minor compositional differences among the radial locations (Figure 13). The quantitative compositional results are summarized in Table 5. The minor differences were comparable to the reported analytical uncertainties and did not exhibit a clear monotonic radial pattern. Accordingly, no systematic radial variation in the principal measured metallic elements was identified within the resolution of the applied methods.

### 3.7. Corrosion Response

**Figure 14:**
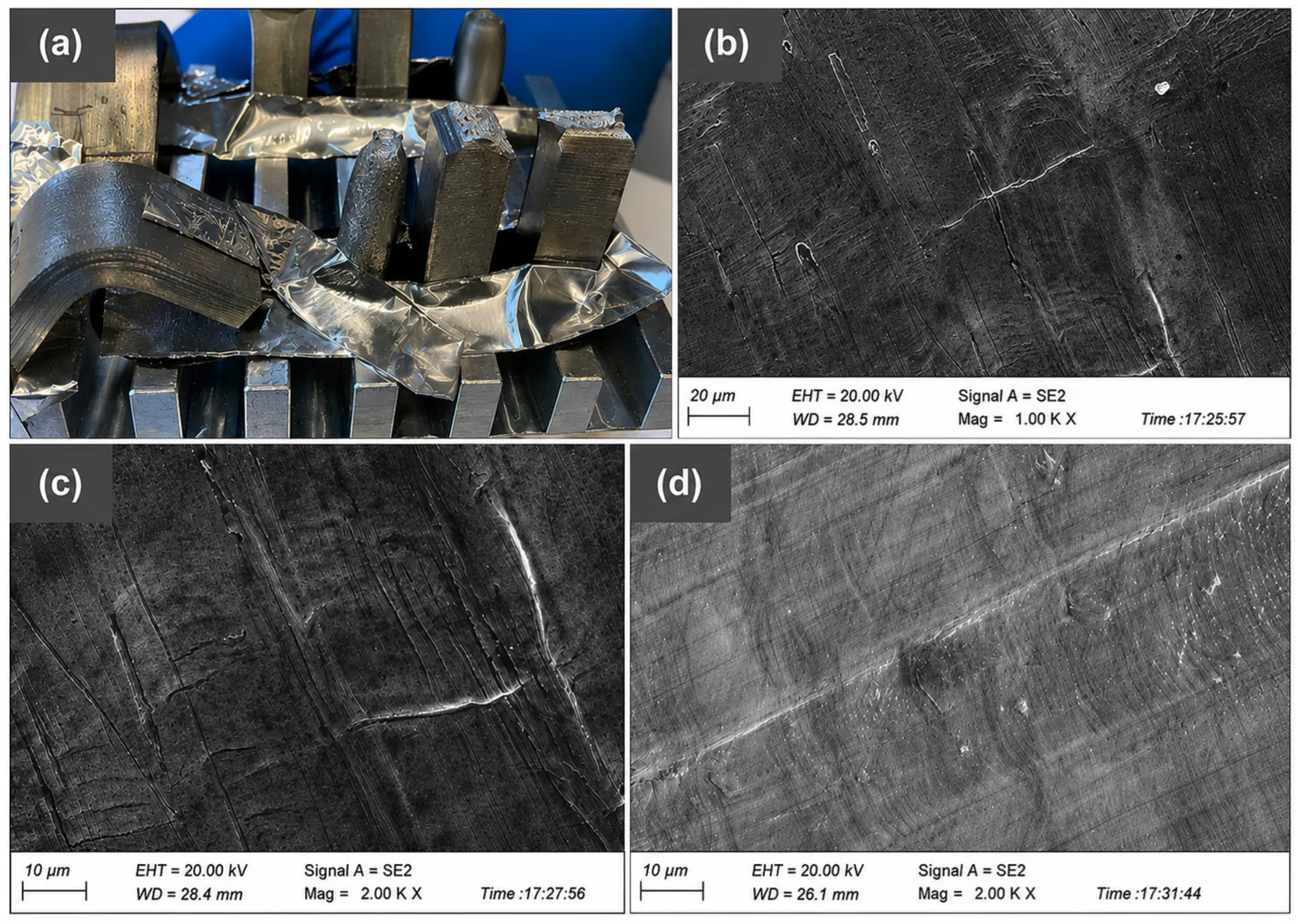
(**a**) Representative post-exposure bend specimens following ASTM A262 Practice E (Strauss) testing; (**b**–**d**) representative SEM micrographs of the bent specimens from the surface, half-radius, and geometric-center regions, respectively.

**Figure 15:**
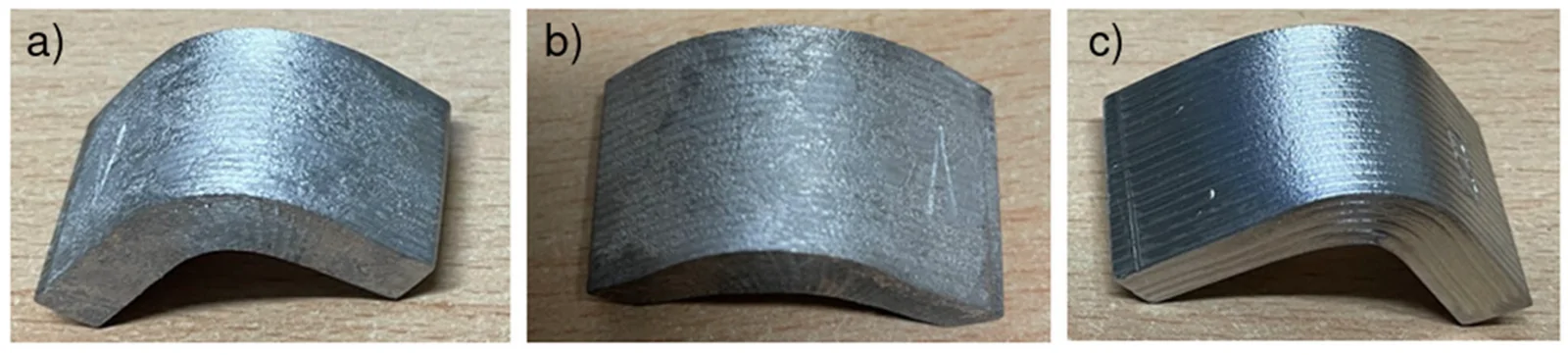
Macroscopic post-test appearance of specimens from the (**a**) surface, (**b**) half-radius, and (**c**) geometric-center regions following exposure according to ASTM A262 Practice E (Strauss test) and subsequent 180° bending.

SEM examination of the bent specimens revealed deformation bands, slip traces, and localized shear features, but no intergranular cracking, grain-boundary separation, or preferential grain-boundary dissolution in the examined regions (Figure 14). Macroscopic examination likewise revealed no visible evidence of intergranular attack following ASTM A262 Practice E exposure and subsequent bending (Figure 15). The qualitative observations are summarized in Table 6.

### 3.8. Vickers Microhardness

**Figure 16:**
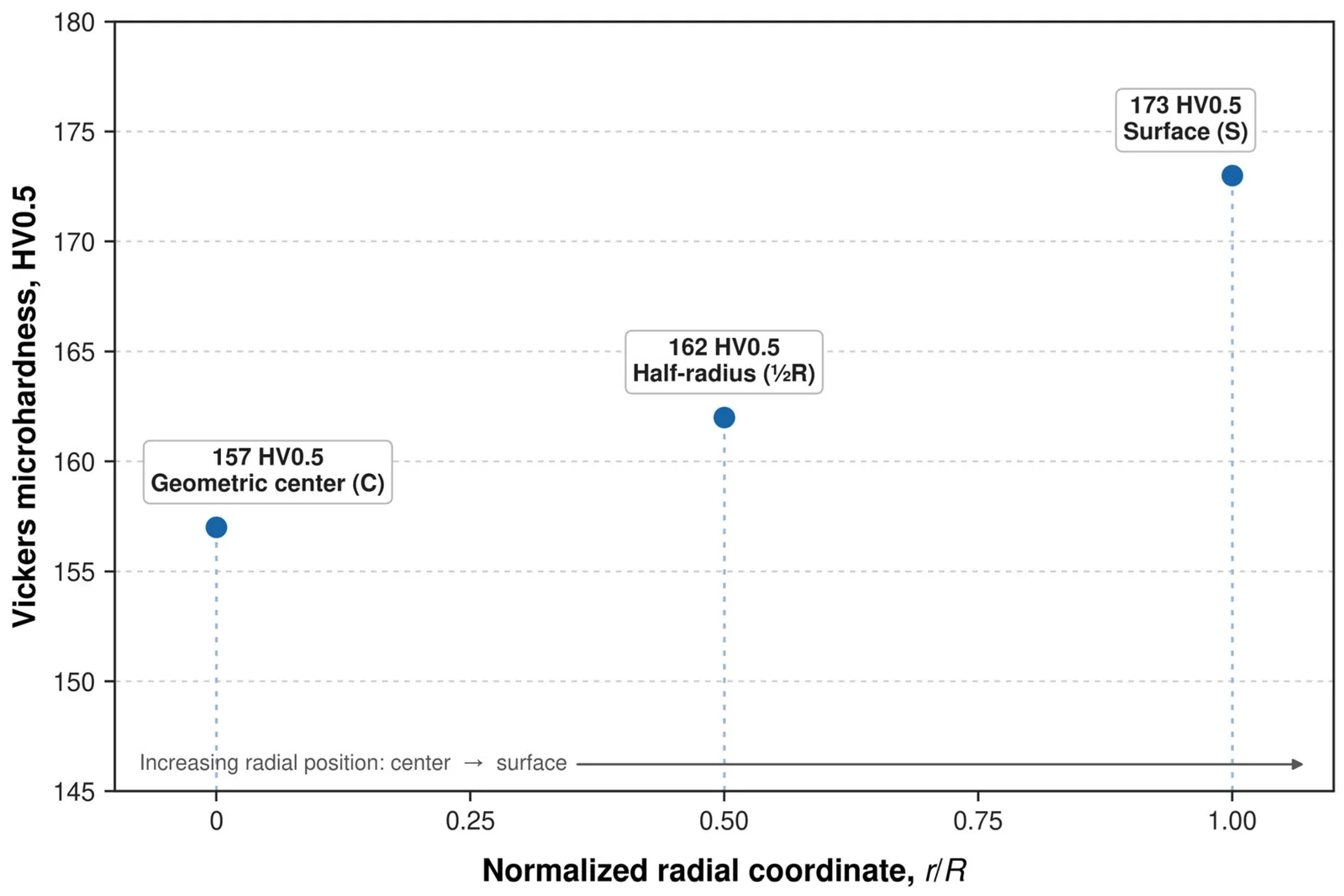
Vickers microhardness values recorded as a function of normalized radial position, *r*/*R*, in the large-section AISI 316 stainless-steel component. Measurements correspond to the geometric center (*r*/*R* = 0), half-radius (*r*/*R* = 0.50), and surface (*r*/*R* = 1.00) regions. Symbols represent the instrument-displayed HV0.5 value at each investigated radial position. Dashed vertical lines are visual guides and do not represent error bars.

Table 7 and Figure 16 summarize the through-section Vickers microhardness results. The instrument-reported values decreased monotonically from 173 HV0.5 at the surface to 162 HV0.5 at the half-radius position and 157 HV0.5 at the geometric center. The center value was approximately 9.2% lower than the surface value. This spatial ordering was consistent with the yield-strength results, although the relative surface-to-center difference was smaller for microhardness. The hardness values therefore provide complementary localized evidence of through-section mechanical-property variation, with lower measured hardness toward the component interior. However, because measurement-level data and associated uncertainties were unavailable, the results are interpreted descriptively rather than as a statistically established relationship. The original instrument records are provided in Figure S5.

## 4. Discussion

The investigated Ø341 mm AISI 316 component exhibited measurable through-section variations in grain size and mechanical properties. The reported mean grain size increased modestly from the surface toward the center, while mean yield strength was 36.3% lower in the nominal central-region tensile group (*r*/*R* = 0.23) than in the near-surface group (*r*/*R* = 0.93). Although the component remained in the furnace for approximately 14.5 h beyond the prescribed holding period, the absence of a conventionally treated control component prevents the observed variations from being attributed specifically to the extended furnace residence. Furthermore, the furnace-zone records do not establish the internal thermal history of the component; therefore, the contribution of through-section thermal-history differences could not be determined directly. The descriptive regression showed a close correspondence between mean yield strength and radial position across the three sampled levels, but this case-specific trend should not be interpreted as a generally predictive material relationship.

At the nominal-group level, lower mean yield strength toward the component interior was accompanied by larger reported mean grain size and lower instrument-reported microhardness. Microhardness decreased from 173 HV0.5 at the surface to 162 HV0.5 at half-radius and 157 HV0.5 at the geometric center, representing a surface-to-center reduction of approximately 9.2%. This spatial ordering was consistent with the yield-strength results, although the relative hardness difference was smaller. The microhardness values therefore provide complementary localized observations consistent with a spatially varying mechanical response across the component section. However, because the individual indentation data and associated uncertainties were unavailable, the hardness results are interpreted descriptively.

According to the Hall–Petch relationship, yield strength commonly decreases with increasing grain size [36,37]:

*σ*_y_ = *σ*_0_ + *k_y_d*^−1/2^ (1)

where *σ*_y_ is the yield strength, *σ*_0_ is the lattice-friction stress, *k_y_* is the Hall–Petch strengthening coefficient, and *d* is the mean grain size. The inverse group-level correspondence between the reported grain-size values and measured yield strength was qualitatively consistent with established grain-size-strengthening behaviour. However, the Hall–Petch relationship was not fitted because only three nominal radial groups were available, the grain-size and tensile measurements were not obtained at identical radial coordinates, and the underlying grain-size measurements could not be retrospectively verified. Prolonged high-temperature exposure in austenitic stainless steels may promote grain growth, recovery, recrystallization, and other microstructural changes capable of reducing strength and hardness [11,13,38]. The approximately 6.3% increase in reported mean grain size from the surface to the center coincided with a substantially larger, 36.3% reduction in mean yield strength between the near-surface and nominal central-region tensile groups.

To assess the magnitude of the possible grain-size contribution to this difference, a first-order Hall–Petch estimate was performed. Subtracting the Hall–Petch expressions for the surface and central regions eliminates the lattice-friction term and gives:

(2) $$\Delta \sigma_{\mathrm{HP}}=k_{y}\left(d_{S}^{-1/2}-d_{C}^{-1/2}\right)$$

where $d_{S}$ and $d_{c}$ are the archived mean grain sizes, expressed in µm, at the surface and geometric center, respectively. A literature coefficient of ${k_{y}=325\mathrm{MPa}.µm}^{1/2}=$ 0.325 MPa.m^1/2^, was adopted from Astafurov et al. [37] for 316L-type austenitic stainless steel, accounting for the unit correction published in the associated corrigendum [37]. Substituting the archived grain sizes of 286 and 304 µm gives:

(3) $$\Delta \sigma_{\mathrm{HP}}=325(286^{-1/2}-304^{-1/2})\;\approx 0.58\mathrm{MPa}$$

The measured difference in mean yield strength between the nominal surface and central-region tensile groups was 132.0 MPa (363.3 − 231.3 MPa). Thus, the estimated Hall–Petch difference represents approximately 0.44% of the measured strength difference (0.58/132.0 × 100).

However, Astafurov et al. [37] investigated a 316L-type steel with grain sizes of approximately 3–73 µm, whereas the archived values in the present study range from 286 to 304 µm. Therefore, application of the literature coefficient involves extrapolation beyond its experimentally investigated grain-size range. Furthermore, the archived grain-size measurements could not be independently verified, and the central metallographic and tensile measurements were not obtained at identical radial coordinates (*r*/*R* = 0 and 0.23, respectively). Accordingly, the calculation represents an illustrative first-order magnitude estimate rather than a material-specific prediction or quantitative validation of the Hall–Petch relationship. Under these assumptions, the modest reported grain-size variation cannot account for the substantially larger measured yield-strength difference through conventional Hall–Petch strengthening alone. Other unmeasured factors, including dislocation substructure, recovery or recrystallization state, crystallographic texture, residual stress, and prior forging history, may also have contributed. Accordingly, the observed correspondence is restricted to a qualitative comparison among nominal sampling groups. It does not constitute a quantitative validation of the Hall-Petch relationship or establish a causal relationship between grain size and yield strength.

The predominantly ductile fracture morphology was consistent with the tensile behavior, for which mean elongation after fracture ranged from 56.0% to 62.8% across the sampled groups. No transition to a predominantly brittle or intergranular fracture mode accompanied the reduction in strength toward the component interior. Although local variations in dimple size, void distribution, and plastic-flow features were observed, the dominant fracture mechanism remained ductile in the examined specimens. These observations indicate that the radial strength variation occurred without a corresponding change in the overall fracture mode.

The elemental compositions measured by SEM–EDS and handheld XRF were comparable among the surface, half-radius, and central-region sampling locations. The minor differences were comparable to the reported analytical uncertainties and did not exhibit a clear monotonic through-section pattern. The compositional results therefore do not support variation in the measured alloy chemistry as an explanation for the mechanical-property distribution. Similarly, ASTM A262 Practice E testing revealed no evidence of sensitization-related intergranular attack in the examined specimens under the applied experimental conditions.

Taken together, the tensile and microhardness results showed lower measured strength and hardness toward the component interior, while the archived grain-size values showed a modest increase from the surface toward the center. No corresponding monotonic variation in the principal measured alloying elements, transition to brittle fracture, or intergranular attack was detected. The observed property distribution may reflect the combined influence of thermal history and prior processing; however, their individual contributions could not be resolved from the available dataset. Higher-resolution characterization methods, such as electron backscatter diffraction and transmission electron microscopy, would be required to investigate crystallographic texture, local misorientation, dislocation substructure, and possible recovery or recrystallization effects. Interpretation remains constrained by the absence of a conventionally treated control component and direct measurements of the internal component temperature.

From an engineering perspective, this case illustrates that closely corresponding furnace-zone temperatures do not necessarily establish a uniform internal thermal history or material condition in a large-section component. Assessment of substantial industrial heat-treatment deviations may therefore benefit from coordinated through-section mechanical and microstructural characterization, supplemented, where practicable, by direct internal-temperature measurements or validated numerical thermal analyses.

### Limitations of the Study

First, this investigation was based on a single Ø341 mm AISI 316 component that experienced extended furnace residence during industrial solution annealing. No conventionally treated control component with an equivalent material and manufacturing history was available. Consequently, the findings are case-specific, and the study cannot determine whether the measured radial differences existed before the deviation or were modified by it.

Second, tensile testing covered only three unique gauge-center coordinates: *r*/*R* = 0.23, 0.50, and 0.93. Although four specimens were tested at each coordinate, they represent within-position spatial replicates rather than independent radial levels. The regression was therefore fitted to three group means and, with one residual degree of freedom, provides only a descriptive summary rather than a statistically validated continuous relationship.

Third, the characterization methods were not pointwise co-located at all nominal radial groups. Most notably, central-region tensile specimens represented (*r*/*R* = 0.23), whereas central metallography and microhardness measurements were performed at (*r*/*R* = 0). Structure-property comparisons are therefore restricted to the nominal-group level. In addition, only instrument-displayed microhardness values were retained; individual indentation results and associated uncertainties were unavailable.

Fourth, the internal component temperature was not measured directly. Furnace-zone records characterize the monitored furnace environment but do not establish the internal thermal history of the component. Therefore, it could not be determined whether appreciable internal heat retention or radial temperature gradients developed, or whether either phenomenon influenced the measured through-section property distribution. Furthermore, the selected microscopy fields and SEM–EDS and handheld XRF measurements cannot exclude localized microstructural, nanoscale, or compositional variations. Carbon and possible nanoscale carbide precipitation were not reliably characterized.

Finally, corrosion susceptibility was evaluated using ASTM A262 Practice E under accelerated laboratory conditions. Testing was limited to one specimen from each radial position and was therefore interpreted qualitatively. Although no intergranular attack was detected in the examined specimens, this test does not provide a comprehensive assessment of general corrosion, pitting, crevice corrosion, stress-corrosion cracking, or long-term service performance. Additional testing under representative service conditions would be required to evaluate these corrosion modes.

## 5. Conclusions

This study characterized the microstructure, mechanical properties, elemental composition, and ASTM A262 Practice E corrosion response of a 341 mm-diameter AISI 316 stainless-steel component that remained in the furnace for approximately 22.25 h in total, including approximately 16 h at or near the solution-annealing temperature. The archived mean grain size differed modestly across the sampled radial positions, with values of 286 ± 8 µm at the surface, 292 ± 7 µm at half-radius, and 304 ± 10 µm at the center.

- Mean yield strength at the central-region sampling group was 36.3% lower than at the surface, with measured values of 231.3 ± 2.4 and 363.3 ± 21.7 MPa, respectively. Mean ultimate tensile strength was likewise lower at the center than at the surface, with corresponding values of 505.5 ± 6.4 and 616.3 ± 2.8 MPa.
- Mean Vickers microhardness was 173 HV0.5 at the surface, 162 HV0.5 at the half-radius position, and 157 HV0.5 at the center, showing the same radial ordering as the measured strength values. Because only three unique tensile radial coordinates were investigated, the fitted yield-strength relationship should not be interpreted as a statistically validated continuous radial relationship.
- SEM–EDS and handheld XRF analyses did not reveal systematic radial differences in the principal measured metallic elements. Thus, the results did not support detectable bulk compositional variation as an explanation for the measured mechanical-property differences.
- ASTM A262 Practice E testing detected no sensitization-related intergranular attack in specimens from any of the three sampled radial positions under the investigated test conditions.

Overall, the investigated forging showed substantial through-section differences in strength and microhardness despite modest-reported grain-size variation. These findings demonstrate the importance of through-section assessment following industrial heat-treatment deviations. However, without a conventionally treated control or direct internal-temperature measurements, the origin of these differences remains unresolved, and the findings neither demonstrate a benefit from prolonged solution annealing nor justify its adoption as an alternative treatment for AISI 316.

## Figures and Tables

**Table 1:** Nominal sampling-group designations and actual tensile gauge-center coordinates.

Sample Label	Radial Position	Nominal *r*/*R*	Gauge-Center Distance, *r* (mm)	Actual Tensile Gauge-Center *r*/*R*
A	Surface	1.0	158.00	0.93
B	Half-radius	0.5	85.25	0.50
C	Central region	0.0	40.00	0.23

Note: All central-region specimens were located at *r*/*R* = 0, except for the tensile gauge samples at *r*/*R* = 0.23.

**Table 2:** Radial variation in grain size and ASTM grain-size number.

Position	*r*/*R*	Figure	Grain Size, *d* (µm)	ASTM *G*	Reported Uncertainty (µm)
Surface	1	Figure 7a	286	0.70	±8
Half-radius	0.5	Figure 7b	292	0.64	±7
Center	0	Figure 7c	304	0.52	±10

**Table 3:** Individual tensile test results for the three radial sampling groups.

Radial Sampling Group	Specimen	*σ*_y_ (MPa)	*σ*_UTS_ (MPa)	*A* (%)
Surface region (S)	S1	361	619	58
	S2	358	613	56
	S3	393	618	55
	S4	341	615	55
Half-radius region (12R)	R1	280	529	54
	R2	288	533	60
	R3	269	596	59
	R4	305	625	58
Central region (C)	C1	231	505	63
	C2	228	497	64
	C3	233	508	62
	C4	233	512	62

Specimen numbers 1–4 correspond to the nominal North, East, South, and West reference orientations, respectively. For the surface and half-radius groups, these labels denote circumferential sampling directions. For the central-region group, the tensile-specimen gauge centers were located at *r*/*R* = 0.23 rather than at the geometric center (*r*/*R* = 0) because of the specimen geometry. The four specimens at each radial coordinate were treated as within-position spatial replicates and not as independent radial levels.

**Table 4:** Mean yield strength at the three radial levels and descriptive linear-fit statistics.

Radial Group	*r*/*R*	*n*	*σ*_y_ (MPa)	Fitted Mean (MPa)	Residual (MPa)
Central region	0.23	4	231.3 ± 2.4	232.6	−1.3
Half-radius	0.50	4	285.5 ± 15.2	283.3	+2.2
Surface	0.93	4	363.3 ± 21.7	364.1	−0.8

**Table 5:** SEM–EDS and handheld XRF elemental compositions at the investigated radial positions (wt.%).

Radial Position	Method	Fe	Cr	Ni	Mo	Mn	Cu
Surface (S)	XRF	68.34 ± 0.74	16.67 ± 0.37	10.50 ± 0.49	2.19 ± 0.07	1.98 ± 0.30	0.320 ± 0.14
	SEM–EDS	67.7 ± 1.3	16.7 ± 0.6	9.7 ± 0.6	2.5 ± 0.5	1.2 ± 0.4	—
Half-radius (12R)	XRF	68.41 ± 0.58	16.82 ± 0.30	10.33 ± 0.40	2.13 ± 0.05	1.92 ± 0.25	0.290 ± 0.11
	SEM–EDS	68.0 ± 1.1	17.2 ± 0.6	9.8 ± 0.4	2.6 ± 0.5	1.2 ± 0.5	—
Center (C)	XRF	67.97 ± 0.65	16.76 ± 0.36	10.63 ± 0.48	2.08 ± 0.07	1.74 ± 0.29	0.299 ± 0.14
	SEM–EDS	68.6 ± 0.8	17.6 ± 0.7	9.5 ± 0.5	2.4 ± 0.5	1.4 ± 0.4	—

SEM–EDS results are presented as archived instrument-reported values and associated uncertainties. The number of replicate measurements and the basis used to calculate the SEM–EDS uncertainties were unavailable for retrospective verification. Handheld XRF uncertainties correspond to the instrumental ±2σ values reported by the instrument software. The dash (—) indicates that Cu was not quantitatively reported by SEM–EDS.

**Table 6:** Summary of SEM observations of bend specimens following ASTM A262 Practice E testing.

Radial Position	Dominant Observed Surface Features	Evidence of Intergranular Attack
Surface (S)	Deformation-related markings	None detected
Half-radius (12R)	Localized slip features	None detected
Center (C)	More pronounced deformation-band contrast	None detected

Note: Assessment was qualitative and limited to the examined regions.

**Table 7:** Vickers microhardness values at the investigated radial positions.

Radial Position	*r*/*R*	Instrument-Displayed Microhardness (HV0.5)
Surface (S)	1.0	173
Half-radius (12R)	0.5	162
Geometric center (C)	0.0	157

Note: Individual indentation values and associated standard deviations were not retained in the archived records and are therefore not reported.

## Data Availability

The original contributions presented in this study are included in the article/Supplementary Materials. Further inquiries can be directed to the corresponding author.

## Supplementary Materials

The following supporting information can be downloaded at: https://www.mdpi.com/article/10.3390/ma19214482/s1, Figure S1: Photograph of the 150 -mm -long end section removed from the AISI 316 component before specimen extraction. This section was excluded from the characterization programme to minimize potential end effects; Figure S2. Representative SEM–EDS spectra acquired at the (a) surface (r/R = 1), (b) half-radius (r/R = 0.5), and (c) geometric center (r/R = 0) of the investigated AISI 316 stainless -steel component. Insets show the instrument-reported semi-quantitative elemental concentrations (wt.%), with σ denoting the corresponding instrument-estimated analytical uncertainty; Figure S3. Representative photograph of a round tensile specimen used for mechanical testing. All twelve tensile specimens had the same nominal geometry, with an initial diameter (d_0) of 10 mm, a parallel length (L_c) of 75 mm, an original gauge length (L_0) of 50 mm, and an initial cross-sectional area (A_0) of 78.5 mm^2^; Figure S4. Original machine-generated engineering stress–strain plots retained in the archived tensile testing records: (a) R3 (half-radius), (b) C1 (central region), (c) R2 (half-radius), (d) R1 (half-radius), (e) S1 (surface region), and (f) S2 (surface region). “MAX” denotes the software-detected ultimate tensile strength, and “Break” denotes the fracture point; Figure S5. Original Vickers microhardness instrument records from the three-indentation measurement sequence at each investigated radial position. Only the instrument-displayed aggregate HV0.5 value was retained for quantitative reporting; individual indentation values and associated uncertainty measures were unavailable.

## Abbreviations

The following abbreviations and symbols are used in this manuscript:

*A*	Elongation after fracture
AISI	American Iron and Steel Institute
ASTM	ASTM International
C	Nominal central-region sampling group; tensile gauge-center coordinate, *r*/*R* = 0.23
*d*	Average grain size (μm)
*G*	ASTM grain-size number
h	Hours
HV0.5	Vickers hardness measured using a 0.5 kgf test load
IGC	Intergranular corrosion
OM	Optical microscopy
*r*	Radial distance from the geometric center of the component to the centerline of the relevant specimen or measurement location
*R*	Component radius (170.5 mm)
*r*/*R*	Normalized radial coordinate
*R*^2^	Coefficient of determination
S	Nominal surface sampling group; tensile gauge-center coordinate, *r*/*R* = 0.93
12R	Nominal half-radius sampling group; tensile gauge-center coordinate, *r*/*R* = 0.50
SEM–EDS	Scanning electron microscopy–energy-dispersive X-ray spectroscopy
XRF	X-ray fluorescence
*σ*_y_	0.2% proof stress (yield strength)
*σ*_UTS_	Ultimate tensile strength
