# Supplementary material for: Through-Section Characterization of a Large Forged AISI 316 Component: An Industrial Post-Deviation Case Study
Source: Materials (Basel). 2026 Oct 24;19(21):4482. doi: 10.3390/ma19214482 (PMC13764583; doi:10.3390/ma19214482)
Supplement: Supplementary file 1 [file materials-19-04482-s001.zip › materials-4617835-supplementary.pdf]

Supplementary Materials

Through-Section Characterization of a Large Forged AISI 316 Component: An Industrial Post-Deviation Case Study

Jude Emele [1], Aleš Slíva[1], Mahalingam Nainaragaram Ramasamy [1,*], Hasanien Ali Sattar Al Sharif [1] and Jan Dizo [2]

[1] Institute of Transport, Faculty of Mechanical Engineering, VSB Technical University of Ostrava, 17. Listopadu 15, 708 00 Ostrava-Poruba, Czech Republic; jude.emele.st@vsb.cz (J.E.); ales.sliva@vsb.cz (A.S.); hasanien.ali.sattar.al.sharif.st@vsb.cz (H.A.S.A.S.)

[2] Faculty of Mechanical Engineering, University of Zilina, 17. Univerzitna 8215/1, 010 26 Zilina, Slovakia; jan.dizo@fstroj.uniza.sk

* Correspondence: mahalingam.nainaragaram.ramasamy@vsb.cz

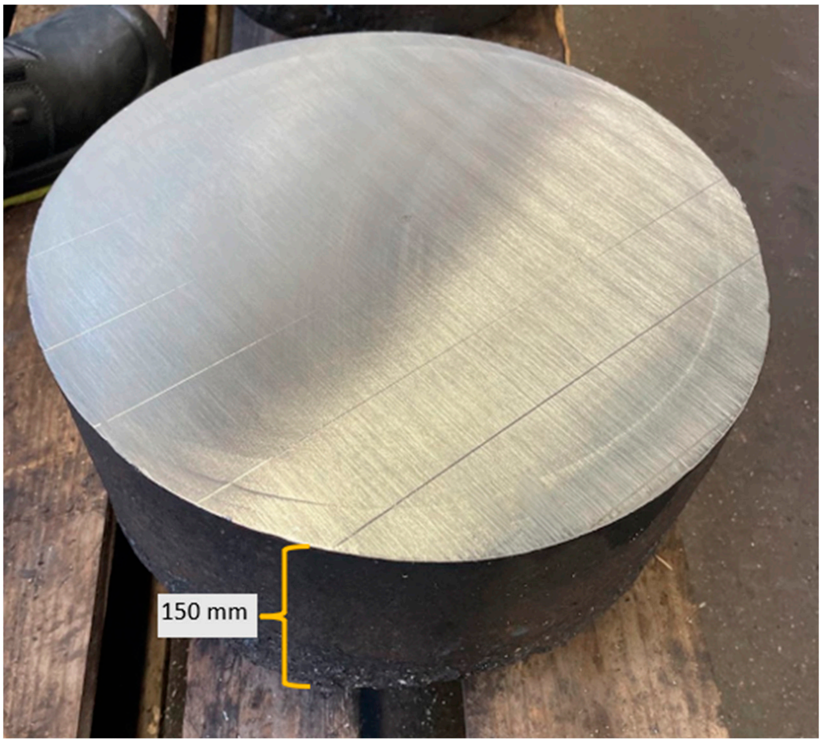

Figure S1. Photograph of the 150 mm long end section removed from the AISI 316 component before specimen extraction. This section was excluded from the characterization program to minimize potential end effects.

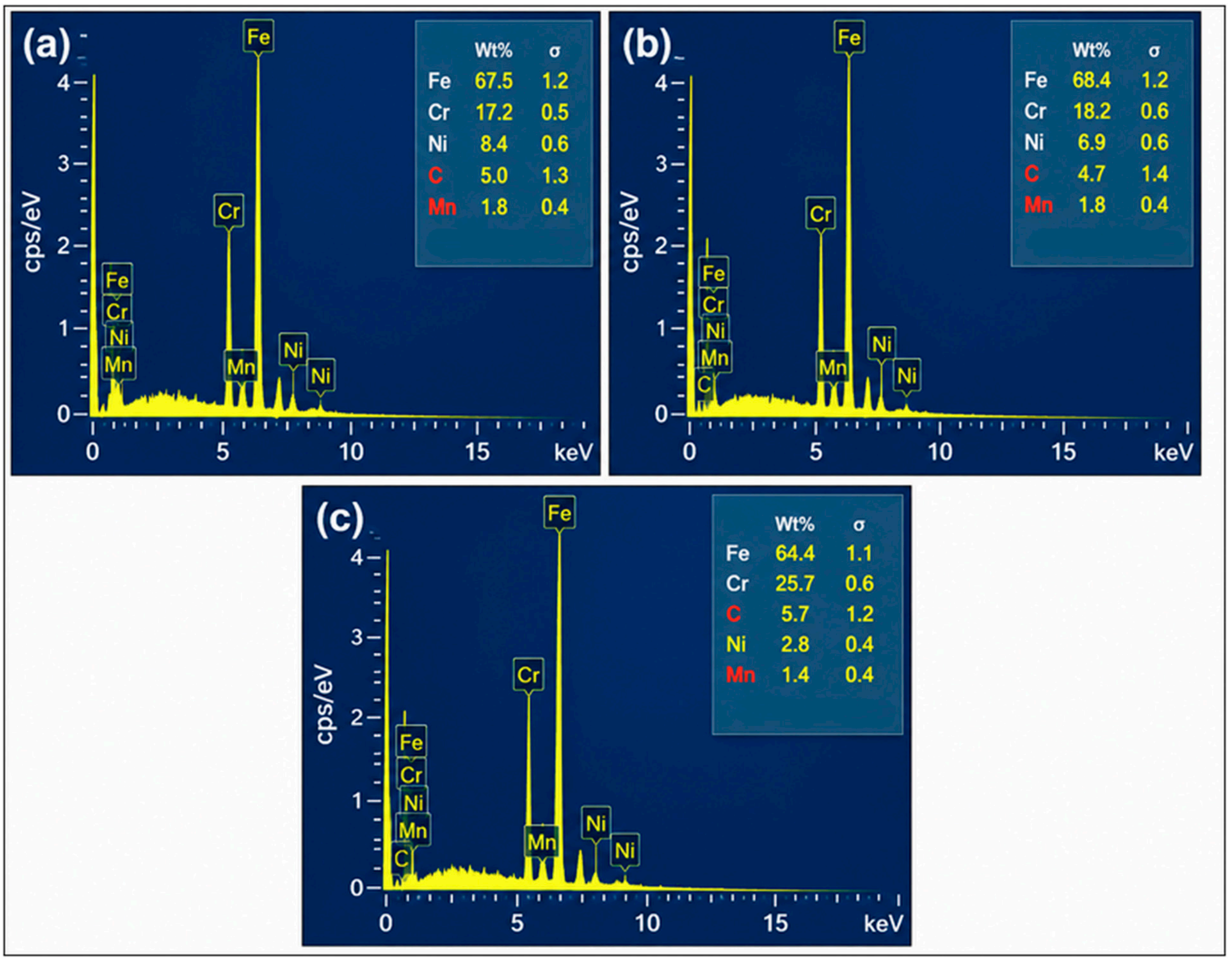

Figure S2. Representative SEM–EDS spectra acquired at the (a) surface ($r/R = 1$), (b) half-radius ($r/R = 0.5$), and (c) geometric center ($r/R = 0$) of the investigated AISI 316 stainless steel component. Insets show the instrument-reported semi-quantitative elemental concentrations (wt.%), with σ denoting the corresponding instrument-estimated analytical uncertainty.

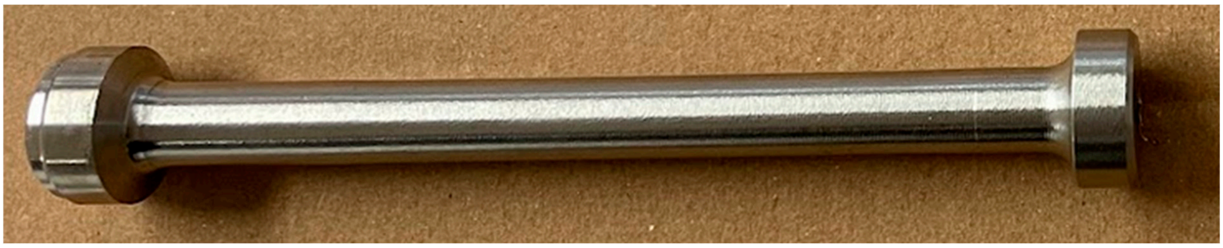

Figure S3. Representative photograph of a round tensile specimen used for mechanical testing. All twelve tensile specimens had the same nominal geometry, with an initial diameter (d_0) of 10 mm, a parallel length (L_c) of 75 mm, an original gauge length (L_0) of 50 mm, and an initial cross-sectional area (A_0) of 78.5 mm^2.

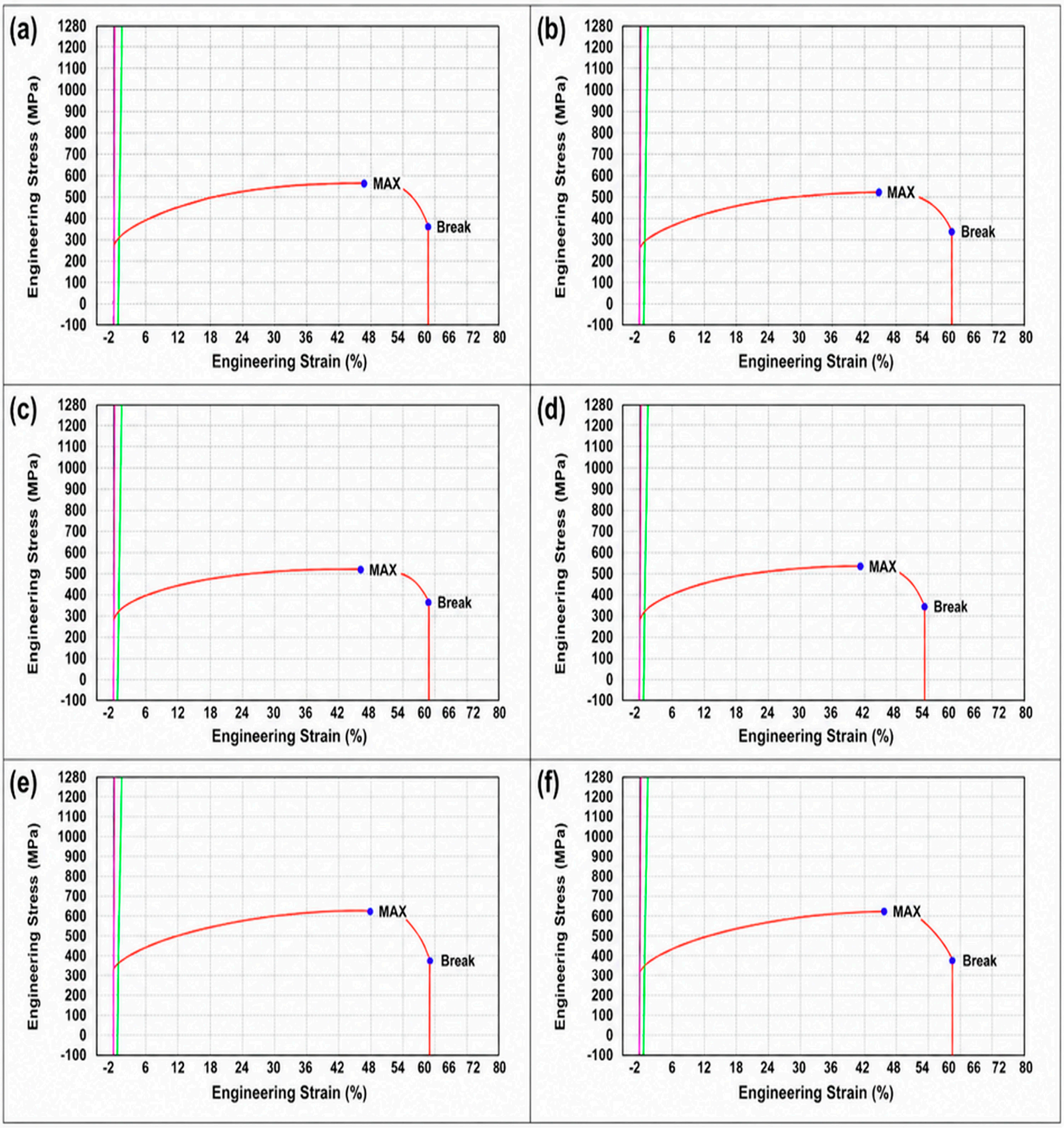

Figure S4. Original machine-generated engineering stress–strain plots retained in the archived tensile testing records: (a) R3 (half-radius), (b) C1 (central region), (c) R2 (half-radius), (d) R1 (half-radius), (e) S1 (surface region), and (f) S2 (surface region). “MAX” denotes the software-detected ultimate tensile strength, and “Break” denotes the fracture point.

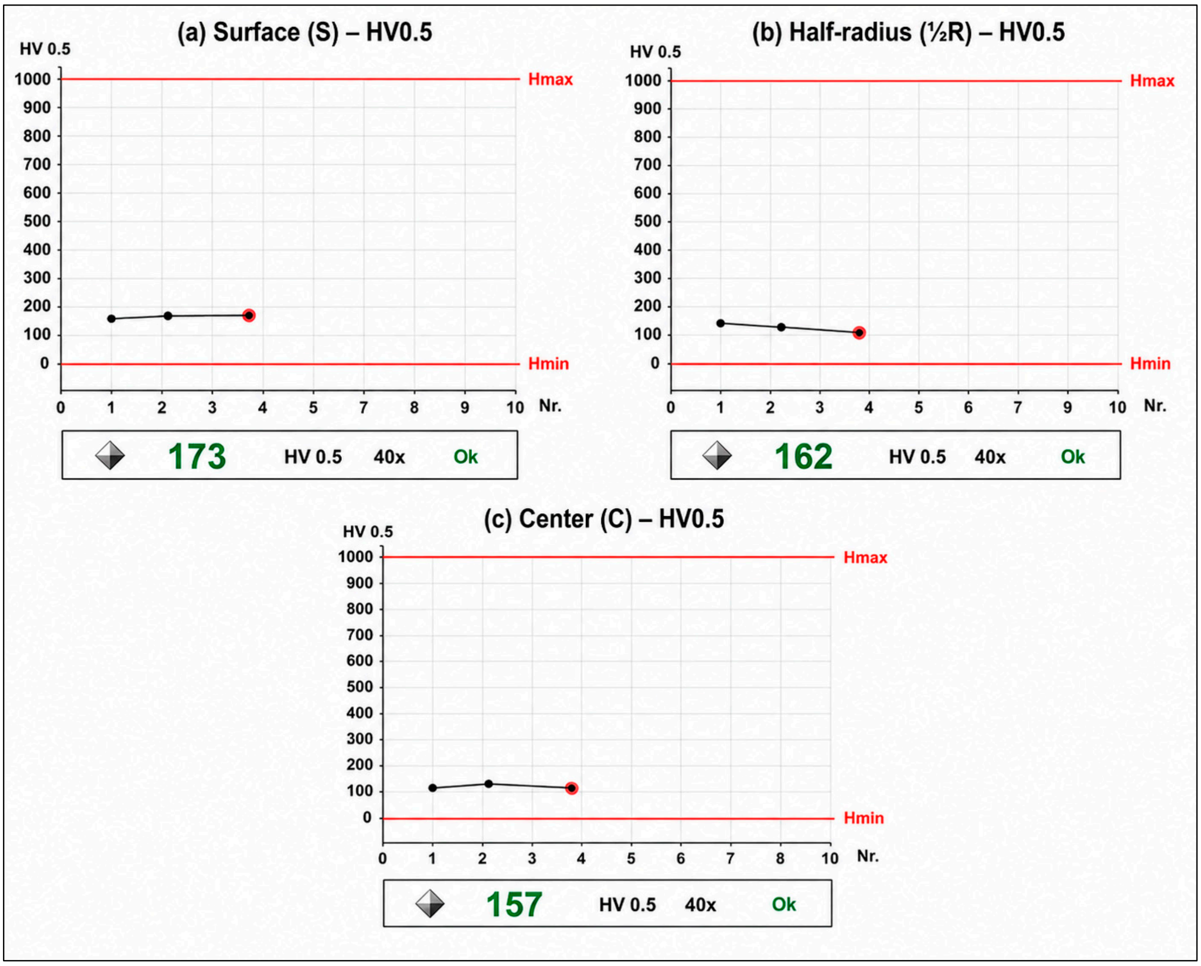

Figure S5. Original Vickers microhardness instrument records from the three-indentation measurement sequence at each investigated radial position. Only the instrument-displayed aggregate HV0.5 value was retained for quantitative reporting; individual indentation values and associated uncertainty measures were unavailable.
